# Distinctive expansion of gene families associated with plant cell wall degradation, secondary metabolism, and nutrient uptake in the genomes of grapevine trunk pathogens

**DOI:** 10.1186/s12864-015-1624-z

**Published:** 2015-06-19

**Authors:** Abraham Morales-Cruz, Katherine C. H. Amrine, Barbara Blanco-Ulate, Daniel P. Lawrence, Renaud Travadon, Philippe E. Rolshausen, Kendra Baumgartner, Dario Cantu

**Affiliations:** Department of Viticulture and Enology, University of California Davis, Davis, CA 95616 USA; Department of Plant Pathology, University of California Davis, Davis, CA 95616 USA; Department of Botany and Plant Sciences, University of California Riverside, Riverside, CA 92521 USA; United States Department of Agriculture - Agricultural Research Service, Crops Pathology and Genetics Research Unit, Davis, CA 95616 USA

**Keywords:** Comparative genomics, Computational Analysis of gene Family Evolution (CAFE), CAZymes, Peroxidases, Secondary metabolism, P450s

## Abstract

**Background:**

Trunk diseases threaten the longevity and productivity of grapevines in all viticulture production systems. They are caused by distantly-related fungi that form chronic wood infections. Variation in wood-decay abilities and production of phytotoxic compounds are thought to contribute to their unique disease symptoms. We recently released the draft sequences of *Eutypa lata*, *Neofusicoccum parvum* and *Togninia minima*, causal agents of Eutypa dieback, Botryosphaeria dieback and Esca, respectively. In this work, we first expanded genomic resources to three important trunk pathogens, *Diaporthe ampelina*, *Diplodia seriata,* and *Phaeomoniella chlamydospora*, causal agents of Phomopsis dieback, Botryosphaeria dieback, and Esca, respectively. Then we integrated all currently-available information into a genome-wide comparative study to identify gene families potentially associated with host colonization and disease development.

**Results:**

The integration of RNA-seq, comparative and *ab initio* approaches improved the protein-coding gene prediction in *T. minima*, whereas shotgun sequencing yielded nearly complete genome drafts of *Dia. ampelina*, *Dip. seriata,* and *P. chlamydospora.* The predicted proteomes of all sequenced trunk pathogens were annotated with a focus on functions likely associated with pathogenesis and virulence, namely (i) wood degradation, (ii) nutrient uptake, and (iii) toxin production. Specific patterns of gene family expansion were described using Computational Analysis of gene Family Evolution, which revealed lineage-specific evolution of distinct mechanisms of virulence, such as specific cell wall oxidative functions and secondary metabolic pathways in *N. parvum*, *Dia. ampelina*, and *E. lata*. Phylogenetically-informed principal component analysis revealed more similar repertoires of expanded functions among species that cause similar symptoms, which in some cases did not reflect phylogenetic relationships, thereby suggesting patterns of convergent evolution.

**Conclusions:**

This study describes the repertoires of putative virulence functions in the genomes of ubiquitous grapevine trunk pathogens. Gene families with significantly faster rates of gene gain can now provide a basis for further studies of *in planta* gene expression, diversity by genome re-sequencing, and targeted reverse genetic approaches. The functional validation of potential virulence factors will lead to a more comprehensive understanding of the mechanisms of pathogenesis and virulence, which ultimately will enable the development of accurate diagnostic tools and effective disease management.

**Electronic supplementary material:**

The online version of this article (doi:10.1186/s12864-015-1624-z) contains supplementary material, which is available to authorized users.

## Background

**Fig. 1:**
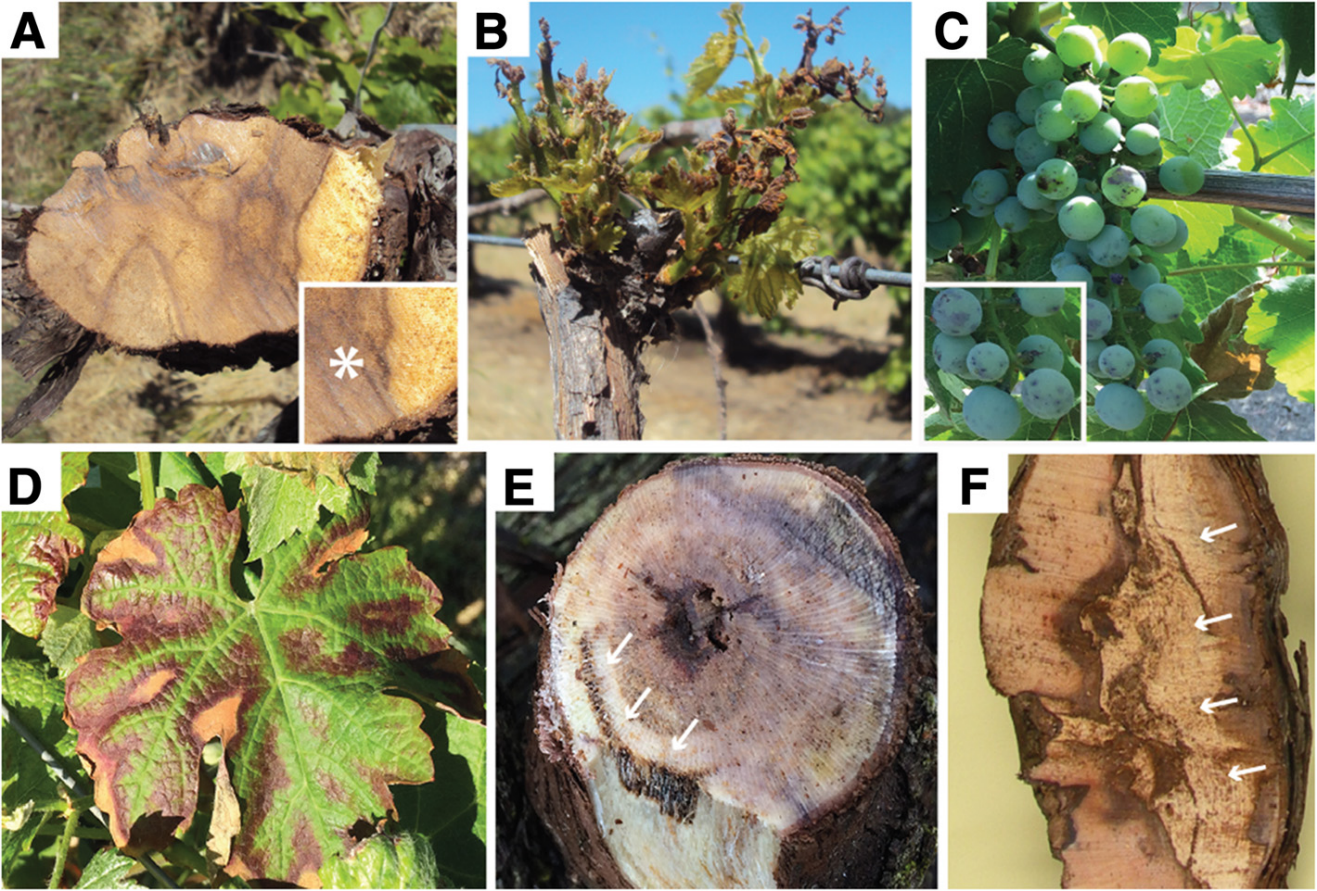
Disease symptoms caused by grapevine trunk pathogens. Images showing the variety of disease symptoms associated with the different ascomycete and basidiomycete pathogens studied in this work. Wood cankers or discoloration reflect the actual fungal infection (**a**, **e**, **f**); this is the localized section of wood where the trunk pathogen resides. The foliar and canopy symptoms that develop distal to the wood canker are due to fungal degradation of the wood (**a**, **e**, **f**) (and subsequent disruption of the flow of water and nutrients) and/or translocation of fungal toxins to the shoots (**b**, **d**). Detailed descriptions of the symptoms are provided in Table 1. (**a**) Cross-section of a diseased vine with an extensive wood canker (*asterisk*) that spans most of the cordon. (**b**) Typical foliar symptoms due to *E. lata* infections. (**c**) Berry spotting (*measles*) associated with Esca. (**d**) Typical Foliar symptoms of Esca in a red variety (*V. vinifera* cv. Cabernet Sauvignon). (**e**) Black streaking (*arrows*) caused by wood colonization of Esca pathogens. (**f**) Discoloration of the wood caused by white-rot fungi

Eutypa dieback, Botryosphaeria dieback, Phomopsis dieback, and Esca are the most important trunk diseases of grapevines (*Vitis vinifera* L.). They are common in vineyards worldwide, where they are responsible for significant reductions in productivity and longevity [1–5]. The causal fungi are taxonomically-unrelated ascomycete species that infect primarily through wounds (namely pruning wounds) and colonize the permanent woody structure of the vine (trunk, cordons, spurs). Damage to the wood from the localized infection (i.e., wood canker) compromises the translocation of water and nutrients throughout the vine, which eventually leads to death of the shoots or the woody tissues that give rise to new shoots [6, 7]. Some symptoms (Fig. 1; Table 1) are shared among different trunk diseases (e.g., cankers; Fig. 1a), whereas others are unique (e.g., foliar symptoms of Eutypa dieback [8], Fig. 1b; fruit symptoms of Esca [9], Fig. 1c). Fungicides can be applied to protect pruning wounds or pruning can be delayed to a time when wounds are less susceptible, but these approaches are neither effective against all trunk pathogens nor are they economically-feasible for all vineyards [6, 10]. Once a vine is infected, the only means of eradicating a trunk pathogen is by physically cutting out infected tissues and retraining new vines. As vines age, they accumulate wood cankers and yield losses build to the point at which management costs outweigh returns [3].

**Table 1:** Fungal species that cause trunk diseases of grapevine and the symptoms associated with each disease

Species	Disease	Symptoms
*E. lata*	Eutypa dieback	V-shaped wood cankers in spurs, cordons and/or trunk (Fig. 1a). Stunted shoots with shortened internodes and small, chlorotic, cupped tattered leaves, showing marginal necrosis and dead interveinal tissue (Fig. 1b). Also, causes general canopy symptoms of shoot dieback and dead spurs.
*Dip. seriata, N. parvum*	Botryosphaeria dieback	V- or irregular-shaped wood cankers in canes, spurs, cordons and/or trunk (Fig. 1a). Also, causes general canopy symptoms of shoot dieback and dead spurs. Can kill buds on infected canes.
*Dia. ampelina*	Phomopsis dieback	V- or irregular-shaped wood cankers in canes, spurs, cordons and/or trunk (Fig. 1a). Also, causes general canopy symptoms of shoot dieback and dead spurs.
		Can kill buds on infected canes.
		Phomopsis cane and leaf spot is thought to be a separate disease, but is also caused by *Dia. ampelina*. The pathogen directly attacks all green tissues of the vine, causing necrotic lesions on the leaves, green stems, and fruit.
*T. minima*, *P. chlamydospora*, *F. mediterranea, S. hirsutum*	Esca disease complex	Black spots (aka “measles”) on berries (Fig. 1c).
		Interveinal chlorosis and necrosis of the leaves (Fig. 1d). Dark spots that form in concentric rings (appears as dark lines in longitudinal section) in the wood (Fig. 1e).
		White-rotted wood, due to secondary infection by basidiomycetes *F. mediterranea* and *S. hirsutum*, sometimes co-occurs in the trunks of vines also infected by the Esca pathogens *T. minima* and *P. chlamydospora* (Fig. 1f). However, these fungi are not responsible for fruit or leaf symptoms of Esca.

The fungi selected for this study are the most widespread and/or virulent species associated with each trunk disease; this table is not an exhaustive list of all trunk pathogens associated with each trunk disease

When colonizing the wood, trunk pathogens are thought to rely on their ability to enzymatically digest the plant cell walls and/or produce toxins to overcome the host’s preformed and inducible defenses [7, 11–14]. Indeed, the chemical structures of secreted toxins, as well as of the products of cell wall degrading activities, have been described for some trunk pathogens [14–16]. For example, degradation of cellulose and xyloglucans, and secretion of oxidases that might participate in the breakdown of wall-bound lignin, were detected in wood colonized by the Eutypa dieback pathogen *Eutypa lata*. *E. lata* is the only trunk pathogen that has been categorized as a wood-decay fungus, specifically a soft-rot, which is the type of wood decay caused by Ascomycetes [14]. Metabolites with phytotoxic properties, such as naphtalenone pentaketides, polyphenols, and extracellular polysaccharides, have been found in the secretomes of Eutypa dieback, Esca and Botryosphaeria pathogens [15]. Although this knowledge is valuable to infer the hypothetical proteins involved in pathogenesis and virulence, there are no documented candidate sequences for any of the potential virulence functions associated with trunk diseases to date. Only scarce molecular genetic information is available for trunk pathogens, compared to other better-studied pathogens of grape (e.g., powdery mildew fungus *Erysiphe necator* [17, 18]*,* Pierce’s disease bacterium *Xylella fastidiosa* [19]). Given that multiple trunk pathogens often co-occur in mixed infections in the vineyard, coupled with the fact that they are taxonomically unrelated, there is a limited understanding of the mechanisms that each trunk pathogen employs to first colonize wood and then cause symptoms [20, 21].

Recent advances in sequencing and genotyping technologies, along with computational tools, offer an unprecedented capability to rapidly generate genomic and transcriptomic resources for plant pathogens [22]. We recently released the first draft genome sequences of the trunk pathogens *E. lata* (causal agent of Eutypa dieback [23]), *Togninia minima* (causal agent of Esca [24]) and *Neofusicoccum parvum* (causal agent of Botryosphaeria dieback [25]). Unique to this study are sequences and analyses of the genomes of other common trunk pathogens (Table 2): *Diaporthe ampelina* (causal agent of Phomopsis dieback)*, Diplodia seriata* (another causal agent of Botryosphaeria dieback), and a Californian isolate of *Phaeomoniella chlamydospora* (another causal agent of Esca). The analysis of functional annotations of their predicted protein-coding genes provided us with a first glimpse of the complex repertoire of potential virulence functions. We identified many genes associated with lignocellulose degradation, toxin production, and nutrient uptake, some of which are known virulence factors in other plant pathogens [26–30]. Comparative analyses also revealed a broad range in the number of members of gene families with potential virulence functions.

**Table 2:** Assembly statistics of the grapevine trunk pathogen genomes analyzed

Species^1^	Disease	Assembly size (Mb)	Scaffolds	Scaffold N50 length (Kb)	Scaffold L50 count	Gene space completeness^2^ (%)	Reference
*Dia. ampelina* (DA912)	Phomopsis dieback^3^	47.4	2,392	132.3	103	94.8	This work
*Dip. seriata* (DS831)	Botryosphaeria dieback	37.1	695	304.2	39	96.0	This work
*P. chlamydospora* (UCR-PC4)	Esca	27.5	702	178.6	50	95.6	This work
*E. lata* (UCR-EL1)	Eutypa dieback	54.0	2,334	68.3	239	93.2	[23]
*T. minima* (UCR-PA7)	Esca	47.5	624	334.6	45	95.2	[24]
*N.parvum* (UCR-NP2)	Botryosphaeria dieback	42.6	1,297	83.6	149	97.2	[25]
*F. mediterranea* (MF3/22)	Esca complex	63.4	1,412	4,291.5	6	96.4	[37]
*S. hirsutum* (FP-91666)	Esca complex	46.5	159	1,799.0	9	96.4	[37]

^1^The name of the isolate that was sequenced is shown in parenthesis

^2^Based on CEGMA analysis [40]. Percentages refer to complete KOGs mapped onto the scaffolds

^3^ *Dia. ampelina* is also the causal agent of Phomopsis cane and leaf spot, a fruit and foliar disease that affects green tissues

Modifications of gene family size, as a result of the differential duplication and deletion of chromosomal regions, have been shown to provide selective advantages and contribute to adaptation in a variety of organisms, including fungi [31, 32]. Gene duplication can be advantageous by increasing the amounts of protein synthesized [18] or by promoting evolutionary novelty of one of the duplicated genes through subfunctionalization or neofunctionalization [33]. In the case of fungal pathogens, variations in gene family size have been associated with the evolution of virulence functions and host adaptation [31, 34–36]. Differential expansion of gene families involved in host cell wall degradation, transport functions, and melanin biosynthesis has been found in pathogenic fungal lineages [35]. Adaptive gene family expansion has also been associated with the shift in host preference from plants to animals in the Onygenales (Ascomycota; Eurotiomycetes) fungi [36].

In this study we employed a stochastic birth and death model to discover gene families that have undergone significant expansion/contraction during the evolution of the trunk pathogens. We first generated a time-calibrated phylogeny using a subset of conserved single-copy protein-coding genes [37] and time of origin estimates from fossil records [38]. The resultant tree was then used to identify those gene families whose size significantly diverged from an estimated random birth-death rate expectation. We identified 90 gene families expanded in the ascomycete trunk pathogens. These gene families were also significantly enriched in putative virulence factors, including cell wall degrading enzymes and genes involved in secondary metabolism. We then applied phylogenetically-aware principal component analysis to detect differences and similarities in the repertoires of putative virulence factors from the significantly expanded gene families.

## Results

### Genome sequencing and gene prediction of *Diaporthe ampelina*, *Diplodia seriata*, and *Phaeomoniella chlamydospora*

**Fig. 2:**
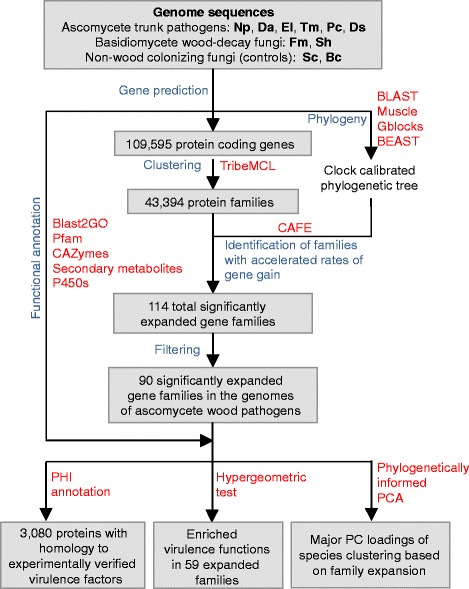
Workflow of the study. Schematic diagram depicting the steps of the analysis (*blue*), the bioinformatics tools applied (*red*), and their output (*gray boxes*)

A diagram describing the experimental workflow of the study is provided in Fig. 2. To perform genome-wide comparisons of virulence factor repertoires across grapevine trunk pathogens, we first expanded the available genomic resources to the haploid genomes of *Dia. ampelina* (isolate DA912)*, Dip. seriata* (isolate DS831), and *P. chlamydospora* (isolate UCR-PC4; Additional file 1: Figure S1). Genome assembly statistics are reported in Table 2 and Table S1 (Additional file 2). On average 98.3 ± 0.58 % of the reads were assembled into 1,263 ± 978 scaffolds (N50 length: 205.04 ± 88.90 Kb; L50 scaffold number: 64 ± 34.2 scaffolds; Additional file 2: Table S1) with total assembly sizes of 47.4, 37.1, and 27.5 Mb for *Dia. ampelina*, *Dip. seriata*, and *P. chlamydospora*, respectively. On average only 4.90 ± 4.67 % discrepancy was observed between the assembled scaffold lengths and genome size estimates based on DNA *k*-mer distributions [39], which suggests that the shotgun sequencing approach delivered nearly complete genomes (Table 2). CEGMA [40] and tRNA analyses also indicated a high degree of completeness of the assembled gene space (Table 2; Additional file 2: Table S1; Additional file 3: Table S2). Transposable elements (TEs) represented only a small fraction of the total assemblies (0.78 ± 0.25 %), confirming observations in ascomycete genomes with similar architecture (Additional file 4: Table S3; [41]). Long-terminal-repeat (LTR) and non-LTR retroelements were the most abundant TEs in the three genomes.

Gene models of the core eukaryotic genes reconstructed using CEGMA were used to train Augustus [42] for ab initio gene discovery. A total of 10,801, 9,398, and 6,986 complete protein-coding genes were identified in the genomes of *Dia. ampelina*, *Dip. seriata*, and *P. chlamydospora*, respectively (Table 2). A similar number of protein-coding genes (7,279) was previously reported for a European isolate of *P. chlamydospora* [43]. Statistics of exon, intron, and intergenic space sizes are reported in Table S4 (Additional file 5) and Figure S2 (Additional file 6). Overall, genes appear to be evenly distributed on the scaffolds of the three genomes, with no evident clustering in gene-rich islands (Additional file 7: Figure S2). Most of the predicted protein coding genes in the three assemblies (94.77 ± 0.88 %) displayed similarity with other ascomycete sequences in the NCBI non-redundant database (BLASTP; e-value < 10^−3^), indicating that the large majority of the predicted genes are *bona fide* protein-coding genes.

### Transcriptome sequencing and improvement of the protein-coding gene models in the *Togninia minima* genome

A hybrid gene prediction approach, using both *ab initio* and RNA-seq based gene discovery, was performed to improve the previously reported gene models of *T. minima* (isolate UCR-PA7 [24]). RNA-seq libraries were prepared from mRNA extracted from *T. minima* colonies growing on different carbon sources to maximize the number of detectable expressed genes (see Methods for details). Ninety-nine percent of the 366 million paired-end RNA-seq reads were assembled *de novo* into 59,610 contigs using Trinity (Additional file 7: Figure S3A; Additional file 8: Table S5 [44]). Open reading frames extracted from the assembled transcripts were used to train Augustus for *ab initio* prediction and, together with 159,358 Uniprot ascomycete curated proteins, were used for evidence-based prediction using the Maker pipeline [45]. As a result, 11,591 complete protein-coding genes were obtained (Additional file 7: Figure S3B; Additional file 9: Table S6), a larger number compared to the 8,926 genes described previously [24]. The integration of RNA-seq with Augustus and Maker gene discovery did not only increase the number of protein-coding genes, but also improved the predicted gene structures, evidenced by wider alignment coverage (Additional file 7: Figure S3C) and greater percent identity when aligned to the proteomes of other ascomycetes (Additional file 7: Figure S3D).

### Annotation of virulence functions in the predicted proteomes

**Fig. 3:**
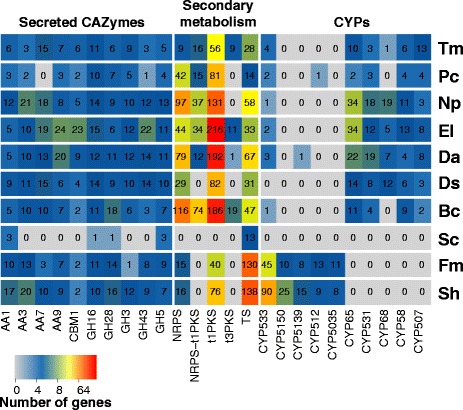
Counts of protein coding genes annotated as secreted CAZymes, P450s, or part of gene clusters involved in secondary metabolism. The heatmap includes only the annotations with the greatest number of genes across all genomes

The proteomes of the six ascomycete grapevine trunk pathogens were annotated, with a focus on key functions likely to be associated with: (i) wood degradation and host colonization, such as carbohydrate-active enzymes (CAZymes), peroxidases, cytochrome P450s; (ii) cellular transporters; and (iii) secondary metabolism, including toxin production (Fig. 3; Additional file 10: Table S7; Additional file 11: Figure S4). Software, databases, and parameters used for annotation are listed in Table 3. The predicted proteomes of the two basidiomycete fungi, *Fomitiporia mediterranea* and *Stereum hirsutum* [37], and two additional ascomycete fungi*, Saccaromyces cerevisiae* and *Botrytis cinerea*, were also annotated using the same pipeline (Fig. 2 and Table 3). *F. mediterranea* and *S. hirsutum* are white-rot fungi that sometimes secondarily colonize grapevines with Esca. They are part of the ‘Esca disease complex’ (Table 1), although they are not considered as the causal pathogens of Esca. The inclusion of *B. cinerea* and *S. cerevisiae* in this analysis allowed us to highlight differences between trunk pathogens and other fungi with different lifestyles. *B. cinerea* is a necrotrophic pathogen of grapevine fruit and foliage, but not woody tissues. *S. cerevisiae* was also chosen as a negative control for the analysis, because it does not cause disease in grapevines and does not feed on plant tissues and, thus, it is expected to have the least number of potential virulence factors. In total, 109,595 protein-coding genes were annotated and the number of proteins in the trunk pathogen genomes were assigned to each functional category (Table 4). All proteins were also surveyed for homologous genes in the Pathogen-Host Interaction database (PHI-base, Fig. 2 and Table 3), which contains experimentally-verified pathogenicity, virulence and effector protein coding-genes from fungi [46].

**Table 3:** Databases and methods used to annotate protein-coding genes in the 10 genomes analyzed

Name of Database	Function	Method	Parameters	Citation
Blast2GO	Protein homology annotation	BLASTP	e-value < 10-3	[122]
Pfam	Conserved domain annotation	HMM	e-value ≤ 1e-3	[123]
CAZYmes Analysis Toolkit (CAT)	CAZyme family annotation	BLASTP	Default	[128]
antiSMASH	Secondary metabolite clusters	HMM	Default (Eukaryotic)	[64]
fPoxDB	Fungal peroxidase family annotation	HMM	e-value ≤ 1e-5	[56]
SignalP 4.1	Presence and location of signal peptide cleavage sites	Neural network	Default (Eukaryotic)	[64]
The Cytochrome P450 Homepage	Cytochrome P450 monooxygenase family annotation	BLASTP	e-value ≤ 1e-5	[129]
Transporter Classification Database	Classification of Membrane Transport Proteins	BLASTP	e-value ≤ 1e-5	[130]
Pathogen-Host Interaction (PHI) database	Experimentally tested genes for pathogen-host interaction	BLASTP	e-value ≤ 1e-5	[46]

**Table 4:** Number of protein-coding genes annotated in each functional category

Species	Total proteins^1^	Secreted CAZymes	Secondary metabolism^2^	P450s	Peroxidases	Transporters	PHI-base^3^
*Dia. ampelina*	10,801 (1136)	432	411	177	57	1,230	1,352
*Dip. seriata*	9,398 (910)	335	142	122	53	1,345	1,120
*P. chlamydospora*	6,986 (410)	142	152	47	33	1,108	913
*E. lata*	11,818 (1224)	484	382	205	51	1,381	1,448
*T. minima*	11,591 (880)	303	118	119	48	1,686	1,389
*N. parvum*	10,470 (1097)	413	353	212	54	1,526	1,384
*F. mediterranea*	11,338 (815)	293	185	127	52	1,171	1,168
*S. hirsutum*	14,066 (1116)	401	279	214	52	1,257	1,288
*B. cinerea*	16,410 (993)	287	464	124	49	1,217	1,326
*S. cerevisiae*	6,717 (377)	65	13	3	22	879	691

^1^Values in parentheses correspond to predicted secreted proteins

^2^Number of genes inside secondary metabolism clusters. Genes from the class “others” are not included in the count

^3^Annotations based on genetic perturbations resulting in reduction of virulence or pathogenicity

CAZymes are proteins with predicted catalytic and carbohydrate-binding domains involved in the degradation, modification, or creation of glycosidic bonds [47]. Because secreted CAZymes can participate in the disassembly of plant cell walls during colonization by pathogens, CAZy annotation, together with prediction of protein secretion, has been used extensively for the identification and classification of cell wall degrading enzymes of plant pathogens [18, 37, 48, 49]. On average 36.6 ± 1.7 % of the putative secreted peptides in the genomes of the eight trunk pathogens were similar to proteins in the CAZy database, indicating a complex repertoire of cell wall degrading functions (Additional file 12: Figure S5; Additional file 13: Table S8; [50]). Glycoside hydrolases (GHs) represented the largest superfamily, ranging from 76 genes in *P. chlamydospora* up to 195 genes in *E. lata*. GH subfamilies involved in the degradation of cellulose and hemicellulose were the most abundant in all genomes (Fig. 3; Additional file 13: Table S8), and included endo-β-1,4-cellulases (GH5), β-glucosidases (GH3), xyloglucan transglucosylase/hydrolases (GH16), and β-xylosidases (GH43) [37, 49, 51]. The highest numbers of GH16 were found in *E. lata*, followed by the Botryosphaeriaceous fungi *Dip. seriata* and *N. parvum.* Similarly, *E. lata* and *N. parvum* were the species with the highest numbers of GH43 and GH5, respectively. Compared to all other trunk pathogens, a greater number of proteins with cellulose-binding domains (CBM1) was found in the *E. lata* genome.

Auxiliary activity (AA) CAZymes are enzymes with redox activity that participate in conjunction with other enzymes in the deconstruction of lignocellulosic material [46]. AA3 genes were particularly abundant in *N. parvum* (21 genes) and *S. hirsutum* (20 genes), whereas the largest number of AA7 genes was found in *E. lata* (19 genes). Among the ascomycete trunk pathogens, AA1 genes were particularly abundant in *N. parvum* (12 genes) and *Dip. seriata* (9 genes). Large numbers of genes encoding AA9 were found exclusively in *E. lata* (24 genes) and *Dia. ampelina* (20 genes; Fig. 3).

P450s have a broad spectrum of functions in fungi, from housekeeping activities, such as synthesis of essential membrane lipids, to synthesis of secondary metabolites, and detoxification of xenobiotic compounds [18, 52, 53]. P450 families were classified in clans according to [54]. Differences in the number of genes belonging to the different P450 classes were observed among species, particularly between the Ascomycetes and Basidiomycetes (Fig. 3 and Additional file 13: Table S8). For example, CYP65s, CYP531s and CYP58s were abundant in the genomes of the ascomycete trunk pathogens (CYP65s: 19.33 ± 13.06; CYP531s: 10.50 ± 7.06; CYP58s: 7.33 ± 3.78), but were not detected in the genomes of the two Basidiomycetes. Conversely, the genomes of *F. mediterranea* and *S. hirsutum* presented large numbers of genes encoding CYP5150s (10 and 25, respectively) and CYP5139s (8 and 15, respectively), which were mostly absent from the ascomycete trunk pathogens (Fig. 3). Also, CYP533 was the clan with the highest number in the Basidiomycetes (67.5 ± 31.82), whereas only a few were detected in the ascomycete trunk pathogens (2.0 ± 1.41) (Fig. 3).

Fungal peroxidases are oxidoreductases involved in numerous and diverse processes, such as lignin breakdown and detoxification of reactive oxygen species produced by the host, which may be associated with virulence [55, 56]. The number of peroxidases identified in the trunk pathogens, based on similarity with proteins in the Fungal Peroxidase database (fPoxDB), ranged from 33 in *P. chlamydospora* to 57 in *Dia. ampelina* (Table 3; Additional file 13: Table S8)*.* Potential class II peroxidases (PODs) were found only in the Basidiomycete white rotters, consistent with the hypothesis that these enzymes evolved after the divergence between Ascomycetes and Basidiomycetes [37].

In plant pathogens, cellular transporters are responsible not only for export of compounds involved in pathogenesis and virulence, but they also may play an essential role in protection against plant defense compounds (e.g., secondary metabolites) during pathogenesis, possibly by exporting host-derived antimicrobial compounds out of the cell [57–59]. In this study, the Transporter Classification Database (Table 3) was used to annotate cellular transporters (Additional file 13: Table S8). The electrochemical potential-driven transporters were the class with the highest number of genes across all species (42.68 ± 8.23 % of all transporters), followed by primary active transporters (27.41 ± 4.28 %). MgtE (TCDB code 1.A.26.1.1) involved in the transport of Mg^2+^ and Co^2+^, PPI (TCDB code 3.A.20.1.1) related to the import of proteins to the peroxisomal lumen, and the Major Facilitator Superfamily (MFS, TCBD code 2.A.1.14.11) were the most abundant transporter families in all trunk pathogens.

### Annotation of secondary metabolism gene clusters

**Fig. 4:**
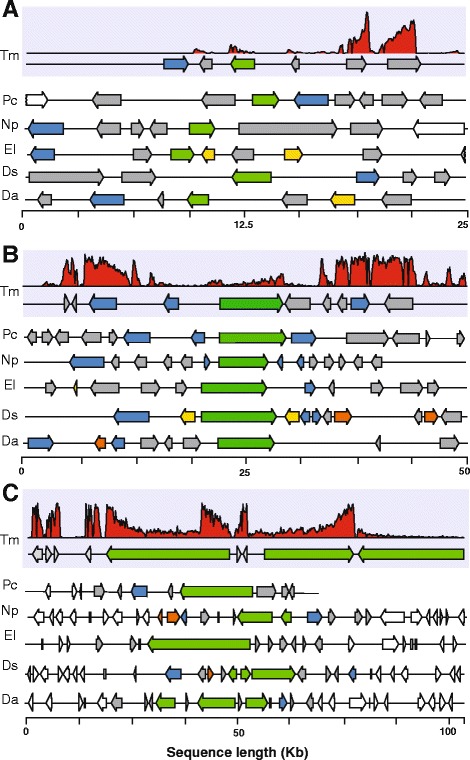
Examples of gene clusters associated with secondary metabolism. Each panel shows loci containing similar set of genes in the ascomycete trunk pathogens associated with synthesis of (**a**) terpenes, (**b**) non-ribosomal peptides and (**c**) polyketides. The loci shown in the figures were not chosen based on their potential orthology across genomes, but solely on the basis of their similar gene cluster composition. Arrows correspond to genes coding for biosynthetic genes (*green*; TS (**a**), NRPS (**b**), and t1PKS), P450s (*yellow*), transporters (*blue*), and FAD-binding proteins (*orange*). Gray arrows correspond to genes predicted to be part of the clusters, but with other annotations, while white arrows correspond to genes outside of the secondary metabolism clusters. Expression levels of *T. minima* genes measured using RNA-seq are reported as mapped read pileups in each panel (first row in each panel)

Products of secondary metabolism, such as toxins and pigments, have been shown experimentally to be important in the development of some symptoms of trunk diseases [15, 60, 61]. Genes involved in the same secondary metabolic pathway are often physically clustered on fungal chromosomes [62]. These gene clusters typically comprise a central biosynthetic gene surrounded by other genes encoding transporters and other enzymes involved in post-synthesis modification of the metabolites, such as cytochrome P450s, dehydrogenases, and FAD binding domain proteins [62–64]. A total of 252 gene clusters involved in the synthesis and secretion of secondary metabolites were identified in all eight trunk pathogen genomes (Additional file 13: Table S8; Additional file 14: Figure S6). The mean cluster size was 37.13 ± 14.30 Kb, containing on average 9.66 ± 4.51 genes per cluster (Additional file 15: Figure S7). The total number of genes related to secondary metabolism per species ranged from 142 in *Dip. seriata* to 411 in *Dia. ampelina. Dia. ampelina* was also the species with the highest diversity of classes, including 11 types of secondary metabolite clusters. The majority of the clusters belonged to type 1 Polyketide Synthases (t1PKS; 40.79 ± 13.61 %), followed by Terpene Synthases (TS, 22.51 ± 7.34 %) and Non-Ribosomal Peptide Synthetases (NRPS; 18.38 ± 8.9 %) in the trunk pathogens (Fig. 3; Additional file 14: Figure S6; Additional file 13: Table S8). Examples of t1PKS, NRPS and TS gene clusters are shown in Fig. 4. In general, *E. lata*, *N. parvum* and *Dia. ampelina* had the highest number of genes related to secondary metabolism, especially t1PKS and NRPS clusters, compared to the other ascomycete trunk pathogens (Table 4). In contrast, t3PKS clusters were found only in *E. lata, T. minima, Dia. ampelina.* TS clusters were particularly abundant in the Basidiomycetes (138 and 130 genes in *S. hirsutum* and *F. mediterranea,* respectively), compared to only 38.50 ± 19.95 genes in the Ascomycete trunk pathogens.

### Estimation of gene family expansion and contraction

**Fig. 5:**
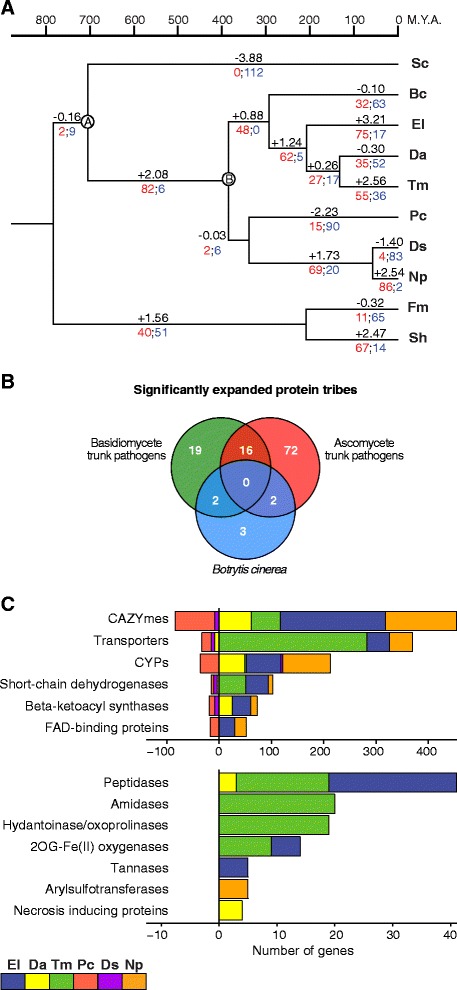
Estimation of gene family expansion and contraction using CAFE. (**a**) Clock calibrated phylogenetic tree showing the number of gene families significantly (*P*-value ≤ 0.01) expanded (red), contracted (blue) and their average pattern (black). (**b**) Venn diagram showing the number of proteins significantly expanded in each group of fungal species. (**c**) Bar plot showing the counts of genes annotated in each group of significantly expanded functional category. Only categories significantly overrepresented (*P-*value ≤ 0.01) in the 90 gene families expanded in the ascomycete trunk pathogens are shown

The Computational Analysis of gene Family Evolution (CAFE; [65, 66]) computer program was utilized to identify gene families that had potentially undergone significant expansion or contraction in the genomes of the analyzed trunk pathogens. CAFE relies on a stochastic birth and death process to model the evolution of gene family sizes for a specified phylogenetic tree using the gene family sizes in the extant species. To apply CAFE, first a clock-calibrated phylogenetic tree was constructed (Fig. 5a) based on the multiple alignments of seventeen conserved peptides previously used to characterize phylogenic relationships across fungi (see Methods and [37]). To strengthen our analysis, 5 additional fungal species with known phylogenetic relationships were included (see Methods and [37]). After GBlocks parsing [67] of the concatenated alignments generated with MUSCLE [68], a total of 8,422 amino acid positions were imported into BEAUti [69]*.* Monophyletic partitions of data were specified and dated following [37] and [38] (see Methods). Branch-length estimation based on fossil records was carried out using the BEAST software package [69]. Branch lengths and tree topology were consistent with previous literature [38]. Our tree also confirmed the topology of recent divergence within the Dothideomycetes and Diaporthales, previously described [37, 38, 70], and the phylogenetically-distant relationship of *P. chlamydospora* and *Dip. seriata*, as described in [71].

To compute the sizes of protein families, the 109,595 proteins of the 10 fungal genomes were clustered into gene families based on sequence similarities (BLASTP; e-value < 1e^−6^) using the TRIBE-MCL algorithm (Additional file 10: Table S7; [72]). Using as input the gene family sizes and the clock-calibrated phylogenetic tree, CAFE identified 114 gene families (9,488 genes) across all fungal species with significantly higher-than-expected rate of gains/losses (*P* ≤ 0.01, Additional file 16: Table S9). Mean gene gains and losses estimated by CAFE for each branch of the phylogenetic trees are shown in Fig. 5a. Among these significantly-expanded gene families, 90 (7,569 genes) were expanded in the ascomycete trunk pathogens, whereas 37 (3,101 genes) were expanded in the two basidiomycetes *S. hirsutum* and *F. mediterranea*. Seventy-two (6,126 genes) and 19 (1,388 genes) gene families were exclusively expanded in the Ascomycete trunk pathogens and in the Basidiomycetes, respectively. Seven gene families (641 genes) were significantly expanded in *B. cinerea* (Fig. 5b). CAFE analysis did not detect any gene family exclusively expanded in *S. cerevisiae*. A list of the genes from the 90 gene families significantly expanded in the ascomycete trunk pathogens, with all annotations carried out in this work, gene family groupings, is provided in Table S10 (Additional file 17).

A hypergeometric test was performed to determine if specific functional categories were significantly overrepresented in the 90 families that were significantly expanded in the ascomycete trunk pathogens (Additional file 18: Table S11). Thirteen of the 37 functional categories were found to be significantly enriched in these 90 families (*P* < 0.01; Fig. 5c; Additional file 18: Table S11). Enriched categories included cell wall degradation, secondary metabolism, protein catabolism, oxidative processes and cellular defense, all of which have been often related to fungal virulence (Fig. 5c). CAZYmes, transporters, and P450s represented the largest functional groups that were overrepresented in the expanded families (Fig. 5c). Expanded CAZyme families were particularly abundant in *E. lata* and *N. parvum*, whereas most of the expanded families of transporters in the trunk pathogens were found in the *T. minima* genome. P450s were the only category enriched in the expanded families of all species, including *B. cinerea* (Additional file 18: Table S11). Genes of the Fe(II)-dependent oxygenase superfamily (2OG-Fell_Oxy), as well as CAZYmes and peptidases, were enriched in the expanded families of all ascomycete trunk pathogens and the basidiomycetes. Among the 72 families expanded exclusively in the ascomycete trunk pathogens, there was a significant enrichment in genes associated with secondary metabolism, such as ketoacyl-synthases and fumarylacetoacetate (FAA) hydrolases (Additional file 18: Table S11). Further evidence in support of a differential expansion of families associated with potential virulence processes comes from our finding that 27.07 % of the 7,569 genes in the expanded gene families in the ascomycete trunk pathogens shared homology with proteins in the PHI-base database (hypergeometric test: *P* < 0.0001).

### Phylogenetically informed principal-components analyses of potential virulence factors in the expanded gene families of ascomycete trunk pathogens

**Fig. 6:**
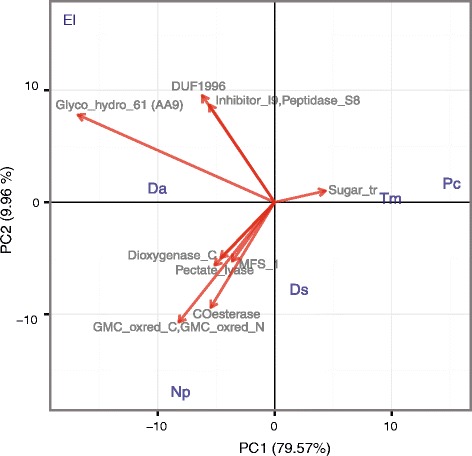
Phylogenetic principal component analysis (PCA) of the expanded gene families grouped by Pfam annotations. Only vectors of the largest loadings are shown

**Fig. 7:**
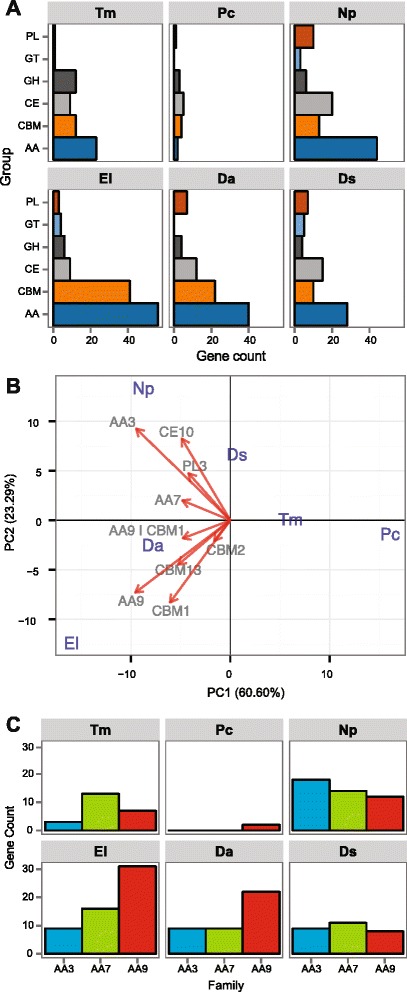
Composition of secreted CAZymes in the 90 significantly expanded gene families of ascomycete trunk pathogens. (**a**) Number of genes in each CAZyme superfamilies. GH: Glycoside Hydrolases, GT: Glycosyl Transferases, PL: Polysaccharide Lyases, CE: Carbohydrate Esterases and AA: Auxiliary Activities. (**b**) Ascomycete trunk pathogens are plotted on the first two principal components based on phylogenetic PCA of CAZymes in the expanded gene families. Only vectors of the largest loadings are shown. (**c**) Bar plot showing the counts of AAs gene in expanded gene families in the ascomycete trunk pathogens

**Fig. 8:**
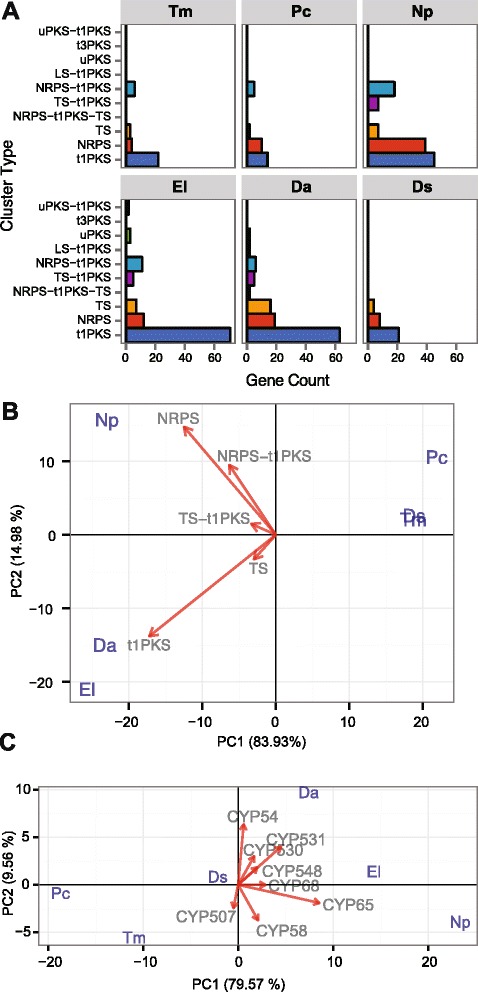
Secondary metabolism related proteins in the 90 significantly expanded gene families of ascomycete trunk pathogens. (**a**) Bar graph of genes divided by secondary metabolism cluster types according to Antismash classification. t1PKS: type 1 Polyketide Synthase, TS: Terpene Synthase, NRPS: Non-Ribosomal Peptide Synthetases, t3PKS: type 3 Polyketide Synthase, uPKS: unusual Polyketide Synthase PKS, LS: Lantipeptide Synthase (LS). (**b** and **c**) Ascomycete trunk pathogens are projected on the first two principal components based on phylogenetic PCA of genes encoding (**b**) proteins involved in secondary metabolism and (**c**) P450s in the significantly expanded gene families. Only vectors of the largest loadings are shown

To visualize the diversity of significantly-expanded families of virulence functions in the ascomycete trunk pathogens and to identify similarities between species, a phylogenetically informed-principal components analysis (PCA) was applied. Members from the 90 expanded gene families in the ascomycete trunk pathogens were grouped into functional categories obtained from the specialized databases mentioned above (Table 3) and PCA was carried out using the Phyl.PCA function, part of the phytools R package [73]. Phyl.PCA takes into account correlations among species due to phylogenetic relatedness, while correcting the correlation matrices for non-independence among observations. Four separate analyses were conducted using the clock-calibrated tree described above and the matrices of the number of genes classified as Pfam (Fig. 6), CAZy (Fig. 7), secondary metabolism (Fig. 8), and P450s (Fig. 8).

PCAs showed that species associated with similar symptoms presented more similar repertoires within the expanded gene families, which often did not correlate with the phylogenetic relationships between species. For example, the two Esca pathogens, *T. minima* and *P. chlamydospora*, were consistently grouped together in all four biplots (Figs. 5–8), suggesting that the two species possess similar virulence repertoires. This is in spite of the fact that *T. minima* is more closely related phylogenetically to *Dia. ampelina*, and *P. chlamydospora* to the two Botryosphaeriaceae *N. parvum* and *Dip. seriata* (Fig. 5a). Nonetheless, these two unrelated Esca pathogens cause similar leaf and fruit symptoms, which are unique compared to symptoms of the other trunk pathogens. Expansions of gene families associated with sugar transport were mostly responsible for the tight clustering of the two Esca pathogens in the biplot based on Pfam annotations (Fig. 6). All four PCAs revealed similar repertoires within the expanded gene families for *E. lata, Dia. ampelina,* and *N. parvum,* which were consistently separated in the biplots, on the other side of the Y-axis (PC2), from *Dip. seriata, P. chlamydospora*, and *T. minima*. Indeed, *E. lata*, *Dia. ampelina*, and *N. parvum* cause similar wood symptoms (v-shaped to irregular-shaped wood cankers), and they all cause shoot dieback and dead spurs. Expansions of families annotated with Pfam Glycoside hydrolase 61 (equal to CAZy AA9), DUF1996, proteinase inhibitor I9 and Peptidase 38 were mostly responsible for the clustering of *E. lata* and *Dia. ampelina* (Fig. 6). Similarities between these two species, particularly in larger numbers of AA CAZymes, mainly AA9s (Fig. 7), genes associated with polyketide synthesis (t1PKS) (Fig. 8b), and P450s genes, primarily CYP54 and CYP531 (Fig. 8c), resulted in their clustering. Clustering of *N. parvum* and *Dip. seriata* was observed when gene counts based on Pfam annotations and CAZYme homologies were analyzed. *N. parvum* showed a distinct expansion of AA3 CAZymes (Fig. 7) and genes encoding secondary metabolic functions, with 49 genes (NPRS + NPRS-t1PKS) compared to 16 ± 7.21 in *E. lata, Dia. ampelina* and *Dip. seriata,* and only 11 ± 1.41 in the two Esca pathogens (Fig. 8). A similar pattern was observed when counts of genes coding for P450s were used as input for phylogenetic PCA (Fig. 8): while PC1 clearly separated the Esca pathogens from the rest, PC2 separated *N. parvum, Dia. ampelina*, and *E. lata*.

## Discussion

In this study we describe the draft genome sequences of three grapevine trunk pathogens, causal agents of Phomopsis dieback, Botryosphaeria dieback and Esca. This genomic information, together with the previously-released draft genome sequences of other important ascomycete trunk pathogens [23–25] and two basidiomycetes associated with Esca [37], provide the genomic resources necessary to begin analyzing the complex repertories of potential virulence profiles of these destructive fungi [20]. All genomes in this study showed a comparable degree of completeness in relation to both genome size estimates, based on *k*-mer distribution and representation of conserved genes [40]. Genome sizes, as well as number of protein-coding genes and repetitive DNA content, were similar to those of other common plant pathogens with a necrotrophic life style, such as *B. cinerea* [41] *Sclerotinia sclerotiorum* [41] and *Colletotrichum* spp. [74]. As observed in [43], *P. chlamydospora* has a relatively smaller genome (and gene content) compared to the other species analyzed. The application of third generation sequencing technologies will help improve these draft assemblies that despite their estimated completeness suffer from limitations due to the use of short reads, which in addition to fragmentary assemblies may include chimeric contigs, erroneous copy numbers and collapsed repetitive regions [75, 76]. We cannot rule out that some protein-coding genes may be missing from the final transcriptomes predicted from the shotgun-sequenced genomes because of (i) inaccessibility of certain genomic regions to Illumina sequencing, (ii) incomplete assemblies, and (iii) possible errors in the *ab initio* gene discovery [76, 77]. Further studies of *in planta* gene expression using RNA-seq may refine the predicted gene models [78]. The effectiveness of integrating transcriptome sequencing with comparative and ab initio approaches for gene prediction is evidenced in this work by the significant improvement of the predicted genes in the genome of *T. minima*.

Functional annotations of the genomes of the 8 trunk pathogens confirmed the complex array of virulence factors that these organisms may utilize during colonization of grapevines. We observed remarkable variation in the number of genes assigned to specific functional categories among the trunk pathogens, which in some instances (and with statistical significance) reflected lineage-specific, gene family expansions and contractions. Gene family expansions result from the retention in a fungal population of duplicated genes, which provide adaptive advantage [35, 79]. Gene duplications can arise from events of genome or chromosomal duplications, TE retroposition, or unequal crossing-over [80]. Gene duplication can lead to functional diversification or increase in protein synthesis, which can play a role in fungal adaptive divergence [81]). An increase in the number of paralogous genes in families associated with virulence and nutrient uptake has been described in the case of obligate parasites [35, 82–86]. Larger family sizes of virulence factors were also found in species with broader host ranges, compared to host-specialized pathogens in the *Metarhizium* genus [87]. Copy number variations (CNVs) within species have also been described not only as a mechanism underlying host adaptation [88–90], but also in the development of resistance to antifungal compounds in fungi [53] (e.g., fungicide resistance in *Erysiphe necator* [18]). The extent and frequency of adaptive CNV in trunk pathogen populations has not been investigated. Nonetheless, as whole-genome sequences of more isolates of each trunk pathogen species become available, comparative approaches can be applied to scan the genomes for CNV loci and determine whether they encompass any of the genes in the significantly-expanded families we identified.

Because natural selection is the major force behind the differences in gene family size between species [66], focusing on families with faster rates of gene gain can help identify functions that may be associated with host adaptation, pathogenicity, or virulence [31]. Among the ascomycete trunk pathogens, we identified 90 gene families that have undergone significant expansion. The expanded families in the ascomycete trunk pathogens were enriched in genes that, at least based on *in silico* annotations, are expected to contribute to virulence and nutrient uptake. The overrepresentation of PHI genes, as well as of secreted CAZymes, P450s, and genes involved in secondary metabolism, supports the role of gene duplication and consequent gene family expansion in the evolution of trunk pathogens. Furthermore, results of phylogenetic PCAs of the sizes of expanded families highlighted similarities between pathogens that did not correspond to their phylogenetic relationships. Instead, pathogens were grouped more often according to similarities in disease symptoms, which suggests there is convergent evolution.

The predicted secretomes of all trunk pathogens encompassed functions that can potentially target all components of primary and secondary plant cell walls (Fig. 3). Unlike pathogens that thrive on pectin-rich tissue, such as *B. cinerea* [49, 91], which possesses high numbers of pectolytic enzymes, overall the ascomycete trunk pathogens showed a wider array of enzymes that target cellulose and hemicellulose, such as endo-β-1,4-cellulases (GH5), β-glucosidases (GH3), xyloglucan transglucosylase/hydrolases (GH16), and β-xylosidases (GH43). As these are wood-colonizing fungi, we might expect their genomes to include a range of genes encoding for wood-degrading enzymes, especially *E. lata*, which is a known soft-rot fungus, and *N. parvum*, which colonizes grapevine wood more rapidly than most trunk pathogens [92]. In agreement with the observation that glucose-rich polymers are degraded in wood colonized by *E. lata* [14], we found significant expansion of genes coding for CAZymes containing the CBM1 domain, a carbohydrate-binding module that strongly binds to crystalline cellulose and that is required for full activity of fungal cellulases. *E. lata* is a soft-rot fungus and has a similar gene family expansion pattern to the white-rot basidiomycete *Phanerochaete chrysosporium*, in which expansion of CBM1s was also found [93]. Indeed, cellulose-degrading systems of some soft-fungi are as advanced as those of typical white-rot fungi [94]. In contrast to *E. lata*, *P. chlamydospora* underwent the least amount of expansion in secreted CAZymes, which is consistent with past findings of no detection *in vitro* of xylanase or cellulases and no visible degradation of lignified cell walls in wood colonized by the latter [11].

The predicted secretomes of the ascomycete trunk pathogens were also rich in auxiliary enzymes (AAs), which catalyze oxidative processes that facilitate the enzymatic disassembly by other CAZymes of recalcitrant plant cell wall components, including lignin [50]. Soft-rot fungi degrade lignin, but to a lesser degree even than brown-rot fungi; they can degrade enough lignin to access other cell wall components that are more efficiently degraded (Worrall et al., 1997). The expansion of AA7s was common in all trunk pathogens, with the exception of *P. chlamydospora*. AA7s are gluco-oligosaccharide oxidases that can oxidize a variety of carbohydrates and can contribute to lignin degradation acting in conjunction with peroxidases [50]. AA3s were expanded the most in *N. parvum*, and AA9s in *E. lata* and *Dia. ampelina*, suggesting that specific oxidative processes are associated with these different dieback pathogens*.* Indeed, the AA3s -- extracellular hemoflavoenzymes and known components of the secretomes of lignocellulose-degrading fungi [95] -- are involved in degradation of cellulose, hemicellulose, and lignin [96]. AA9s are copper-dependent lytic polysaccharide monooxygenases, previously classified as GH61, and are commonly found in genomes of fungal wood decayers. AA9s enhance the breakdown of lignocellulosic material in combination with cellulolytic enzymes by catalyzing the oxidative cleavage of cellulose, which increases substrate accessibility to other CAZymes [50, 97]. *N. parvum* together with *Dip. seriata* also showed expansion of AA1 genes that encode multicopper oxidases, including laccases, which could also participate in lignin breakdown [50].

Secondary metabolites with phytotoxic activity (i.e. toxins) are integral components of the battery of virulence factors of grapevine trunk pathogens [15, 61, 98–102]. Although toxins secreted by some grapevine trunk pathogens have been chemically characterized and tested for virulence, none of the genes involved in their synthesis have been identified to date. In fungi, genes involved in the synthesis and transport of secondary metabolites are typically clustered together with the gene coding for the key biosynthetic enzyme [63, 103]. Large numbers of secondary metabolic clusters were observed in the ascomycete trunk pathogens, mostly associate with the synthesis of (i) polyketides and fatty acid-derived compounds (PKS), (ii) terpenes (TS), and (iii) non-ribosomal peptides and amino acid-derived compounds (NRPS). While NRPS may be responsible for the synthesis of toxic polypeptides [15, 101], clusters centered around the key enzyme polyketide synthase may participate to the production of naphtelenone pentaketide toxins found in *T. minima*, *P. chlamydospora, Dip. seriata* and *N. parvum* [15]. A remarkable expansion of genes associated with non-ribosomal peptides and amino acid-derived compounds was found in *N. parvum*, while the greatest expansions of families involved in polyketide synthesis (t1PKS) were found in genomes of *E. lata* and *Dia. ampelina*. The differences between the Botryosphaeria dieback pathogens *N. parvum* and *D. seriata* in gene counts of these secondary metabolite clusters reflect their different rates of wood colonization [104] and the more rapid rate of wood necrosis caused by the former [105].

Our results also showed that grapevine trunk pathogens possess a large number of P450s as found in other wood-rotting organisms [86, 106, 107]. P450s are crucial components of multiple processes ranging from the biosynthesis of secondary metabolites such as toxins and hormones [52, 108] to degradation and detoxification of antimicrobial plant defense compounds [109]. Interestingly, basidiomycetes and ascomycete trunk pathogens show very distinctive expansions of specific CYPs. Of particular interest are the CYP65s which were more abundant in the ascomycete trunk pathogens (e.g. 34 genes in *N. parvum* and 34 in *E. lata*) compared to *B. cinerea* (11) and were not detected in the two basidiomycete analyzed and *S. cerevisiae.* CYP65s are P450s predicted to participate in pathways of secondary metabolism, including toxin biosynthesis [110].

## Conclusions

As part of this study we expanded the currently available genomic resources for grapevine trunk pathogens and incorporated this information with previously released genomes in a comparative analysis to catalogue genes and gene families with putative virulence functions. The draft genomes and their annotated protein-coding genes presented in this paper provide not only the necessary references for *in planta* expression profiling and whole-genome re-sequencing for genetic diversity and association studies, but also the molecular information required for targeted knock-out mutations, overexpression or gene tagging as protocol for genetic transformation of some of these species are available [111, 112]. Comparisons between *in planta* and *in vitro* transcriptomes will define the expression dynamics of the proposed virulence factors during the interaction with the host. Whole genome re-sequencing of multiple isolates will determine the pattern of sequence polymorphisms [113] and structural variations [18] in pathogen populations and their association with pathogen aggressiveness and host range. The functional validation of these potential virulence factors by reverse genetic approaches will ultimately lead to a more comprehensive understanding of the mechanisms underlying the different grapevine trunk diseases, which will enable the development of more accurate diagnostic tools and novel effective control methods.

## Methods

### Biological material

Vineyards were surveyed for general signs of trunk diseases, including low vigor, stunted canopy, missing spur positions, dead cordons, and overall decline. Samples were collected from cankered, necrotic wood parts observed in cross-sections of vine trunks and arms. Isolates were recovered from the margins of wood cankers. Five pieces of wood, approximately 2 × 5 mm, were cut from the canker margin with a flame-sterilized knife, surface-sterilized in 0.6 % sodium hypochlorite (pH adjusted to 7.2) for 30 s, rinsed twice in sterile distilled water for 30 s, and plated on potato dextrose agar (PDA, Difco, Detroit, MI) amended with tetracycline (1 mg/L). PDA plates were incubated at 25 °C in darkness for several days. Fungal colonies were further hyphal-tip purified. *Dip. seriata* isolate SBen831 (DS831) was recovered from diseased grapevine wood (*V. vinifera*) sampled in Hollister, San Benito Co., California, in June 2011. *Phomopsis viticola* (teleomorph: *Dia. ampelina*) isolate Wolf912 (DA912) was recovered from diseased grapevine wood in the upper trunk of a ‘Seeded Thompson” vine sampled in the USDA-ARS National Germplasm Repository grapevine collection located in Winters, Solano Co., California, in May 2011. *P. chlamydospora* isolate UCR-PC4 was recovered from diseased grapevine wood (*V. vinifera* cv. Flame) from the black necrotic streaks of the wood vascular system sampled in Coachella Valley, Riverside Co., California, in November 2011. Species were confirmed by aligning the internal transcribed spacer (ITS) DNA sequences with their respective type specimens (Additional file 1: Figure S1). ITS sequences were submitted to GenBank and can be found under the following accessions: KP296243 (*Dip. seriata* DS831), KM669964 (*Dia. ampelina* DA912), and KP296244 (*P. chlamydospora* UCR-PC4).

Axenic cultures of isolated DA912, DS831 and UCR-PA7 were grown in PDA (Difco) media. Around 100 mg of mycelia were collected in a 2 ml micro-centrifuge tube and frozen in liquid nitrogen, mycelia were ground while still frozen. DNA extraction was done with a modification of the Cetyltrimethylammonium Bromide (CTAB) protocol [114], beginning with 500 μl of extraction buffer. Concentration and purity of the DNA was measured with Nanodrop 2000c Spectrophotometer (Thermo Scientific). Integrity was checked with an agarose gel.

### Genome sequencing, assembly, and gene prediction

DNA libraries were prepared as described in [18]. Genomic DNA was fragmented with the Bioruptor NGS (Diagenode, USA) in 6–9 cycles of 15 s / 90s (on/off). Libraries were prepared using the KAPA KK8201 library preparation kit (KAPA Biosystems, USA) with approximately 1 μg of fragmented DNA as input. NEXTflex primers were used as barcodes (BIOO Scientific, TX, USA) and size selection was done with the Pippin Prep platform (Sage Science, USA). Final libraries were evaluated for quantity and quality with the High Sensitivity kit in a Bioanalyzer 2100 (Agilent Technologies, CA, USA). Sequencing was carried out on an Illumina HiSeq2500 machine at the DNA Technologies Core at UC Davis. Paired-end reads of 150 bp in length were generated and a median sequencing coverage of 50X, 79X, and 107X was achieved for *Dia. ampelina*, *Dip. seriata*, and *P. chlamydospora*, respectively. Quality trimming (Q > 20) and adapter contamination removal were carried out using sickle (v1.210; [115]) and scythe (version 0.991; [116]), respectively. Assembly of high quality reads was performed using CLC Workbench 6.1. Assembly parameters were optimized to achieve maximal assembly completeness of the gene space estimated using the Core Eukaryotic Genes Mapping Approach (CEGMA) analysis [40]. Contigs with similarity to non-fungal sequences in the complete NCBI nt collection were considered contaminants and were discarded. The assemblathon_stats.pl script [117] was used to compute basic assembly metrics. Genome sizes were calculated from DNA k-mer count distributions [39]. Gene models of the core eukaryotic genes reconstructed using CEGMA were used to train Augustus (v. 2.5.5, [42]) for ab initio gene prediction using default parameters. Predicted Incomplete coding sequences were discarded.

### Transposable elements annotation

Transposable elements were annotated using both ab initio and reference based computational approaches as described in [18]. Ab initio prediction was carried out using RepeatModeler (v1.0.7; [118]). RepeatModeler output (i.e. all identified repeats with the exception of TEs marked as “unknown”) were added to the RepeatMasker database and used as reference for homology-based discovery of interspersed repeats and low complexity DNA sequences using RepeatMasker (v4.0.2; [119]).

### Transcriptome sequencing, *de novo* assembly, and gene prediction (*T. minima*)

In March of 2013 pruned-dormant grapevine canes from *V. vinifera* cv. Cabernet Sauvignon and *V. vinifera* cv. Merlot plants were collected. The canes were cut into 1 cm long fragments, dried at 60 °C for 24 h to reduce water content and ground to fine particles using a Wiley Mini-Mill (Thomas Scientific, NJ, USA). The ground wood (2 % w/v) was mixed with agar (1.5 % w/v) [120]. *T. minima* UCR-PA7 was propagated at 25 °C in PDA and the minimal media with wood described above. The fungus was grown for at least two cycles in the same medium before the actual experiment to avoid a carryover of signals from other media.

Mycelia were collected in a 2 ml micro-centrifuge tube in a sterile environment and were quickly frozen in liquid nitrogen. A stainless-steel bead of 5 mm was added to each tube and ground in the TissueLyser II (Qiagen, CA, USA) at 30 Hz for 30 s with the Teflon adapters pre-cooled to avoid sample thawing. One milliliter of TRIzol reagent (Ambion, USA) was added to the ground mycelia; extraction of total RNA was done following manufacture’s protocol. The RNA was cleaned up with the RNeasy Plant Mini Kit (Qiagen) including the on-column DNase enzymatic treatment. Purity was measured with the Nanodrop 2000 spectophotometer (Thermo Scientific), quantity with the RNA kit of the Qubit 2.0 Fluorometer (Life Technologies, CA, USA) and integrity in an agarose gel.

Libraries were prepared using the Illumina TruSeq RNA sample preparation kit v.2 (Illumina, CA, USA), following Illumina’s protocol (Low-throughput protocol) and barcoded individually. Final libraries were evaluated for quantity and quality with the High Sensitivity kit in a Bioanalyzer 2100 (Agilent Technologies, CA). Libraries were sequenced with HiSeq 2500 as 100 bp paired-ends (Illumina, CA, USA).

Scythe and sickle were used to quality filter and trim the raw reads as described above. *De novo* genome-guided assembly using TopHat (version 2.0.9; [121]) with parameters -r 100 --mate-std-dev 50 --min-intron-length 20 and Trinity (version r20131110; with jaccard_clip option on [44]) was performed as describe in [18]. The perl script transcripts_to_best_scoring_ORFs.pl from Trinity was used to extract putative open reading frames from the assembled contigs. Gene structures of the CEGs identified in the genomic scaffolds by CEGMA together with the *de novo* assembled transcripts were used to train Augustus (v. 2.5.5 [42]) for ab initio gene discovery on repeat-masked scaffolds. MAKER (version 2.8; [45]) was then used to integrate the *ab intio* prediction with homology based gene prediction using the *de novo* assembled transcripts and 159,358 Uniprot ascomycetes curated proteins.

### Gene annotation

Basic annotation of all predicted protein coding sequencing was assigned based on similarity to ascomycete peptides in GenBank with Blast2GO [122] and to conserved domains in the Pfam database [123] (Additional file 10: Table S7). Databases, software and parameters used for the functional annotations are shown in Table 3. The presence of secretion signal peptides was evaluated using SignalP v.4.1 [124]. Proteins were clustered in families based on sequence similarity using BLASTP (e-value < 10^−6^) followed by Markov clustering with TribeMCL [72].

### Construction of a clock-calibrated phylogenetic tree

Twenty-six single copy conserved peptides used in [37] for phylogeny reconstruction and CAFE analysis were extracted for the five Ascomycete species (*Aspergillus niger, Cryphonectria parasitica, Pichia stipitis, Stagonospora nodorum,* and *Trichoderma reesei*) [37]. *S. cerevisiae* orthologs (2S88C reference strain) were downloaded from http://www.yeastgenome.org. All proteins were independently examined using BLASTP (v.2.2.29+) against the proteomes of the 6 ascomycete trunk pathogens and *B. cinerea*. Seventeen protein families were confirmed to have exactly one top hit for all species (Additional file 19: Table S12). Nine families were excluded to avoid including potential paralogs. *S. cerevesiae*, *S. hirsutum*, and *F. mediterranea* orthologs were obtained from [37] and added to the set of orthologous peptides. Alignment of each set of orthologous peptides was carried out with MUSCLE (v. 3.8.31; [68]). Alignments were then concatenated (total of 17,843 positions) and Gblocks (v. 0.91; maximum number of contiguous nonconserved positions allowed = 4, minimum length of a block allowed = 10; [67]) was used to remove ambiguous regions, resulting in an alignment of 8422 amino acids for fifteen species.

Alignments were imported into BEAUti (v. 1.8.0) to prepare for BEAST (v. 1.8.0) analysis. Monophyletic partions of data were specified in BEAUti (Additional file 20: Text S1). The Ascomycota partition was dated using an offset of 400 MY and a log mean of 6.11 following [37] (Node A, Fig. 3a). The Letiomycota partition was dated using an offset of 306 MY with a log mean of 5.57, consistent with [38] (Node B, Fig. 3a). Six MCMC chains were launched using BEAST with length of 1,000,000 (WAG substitution model, 4 Gamma Categories + Invariant sites, Lognormal relaxed clock, Birth-death process). Resulting trees were concatenated with LogCombiner (v. 1.8.0) and loaded into TreeAnnotator (v. 1.8.0) to create a consensus tree (Additional file 21: Figure S8). Branch lengths and tree topology were consistent with previous literature [38]. Although estimated sample size (ESS) values did not reach above 100 (Additional file 22: Table S13), the topology of recent divergence in the Dothideomycetes and Diaporthales partitions, which was not specified in BEAUti, arranged as previously described in [37, 38, 70, 71] and were confirmed by phylogenetic analysis of the same alignments using MrBayes [125] and PhyML [126]. As observed in [37, 38, 70], there is weak statistical support for the taxonomic grouping of *P. chlamydospora* and *Dip. seriata* (Posterior *P =* 0.5145, Additional file 21: Figure S8)*.* Our tree topology confirmed phylogenetic relationships between *P. chlamydospora* and *Dip. seriata* reported in [71].

### Computational analysis of gene family evolution (CAFE)

The tree created with BEAST was trimmed prior to CAFE analysis by removing the five species originally used for branch length calibration. Gene numbers annotated for 8031 tribes, each containing at least one protein in no less than four species, for each of the ten remaining species, along with the tree, were used to run CAFE (v. 3.1; [127]). CAFE was run with default parameters optimizing the lambda parameter (option -s) to 0.000940686 with a *P-*value cutoff of 0.01 (option -p). Viterbi *P*-values were computed for each significant tribe to assess significant expansion or contraction along a specific branch.

### Phylogenetic principal component analysis

The phyl.pca from the R package phytools (www.phytools.org/) was used to compute phylogenetic PCA. The clock-calibrated phylogenetic tree was used to correct for nonindependence among observations. The BEAST tree as well as matrices of Pfam, CAZyme, secondary metabolism, and P450 gene counts were used as input.

### Data access

The whole genome shotgun projects have been deposited in the GenBank database [accession nos.: LAQI00000000 (*Dip. seriata*), LCUC00000000 (*Dia. ampelina*), LCWF00000000 (*P. chlamydospora*)]. The raw reads are available via Sequence Read Archive [accession nos.: SRR1772171 (*Dip. seriata*), SRR1693722 (*Dia. ampelina*), SRR1772173 (*P. chlamydospora*). RNA-sequencing data used in this study have been deposited in the National Center for Biotechnology Information Gene Expression Omnibus (GEO) database, http://www.ncbi.nlm.nih.gov/geo (no. GSE64404).

## Additional files

Additional file 1: Figure S1. Single most likely phylogenetic tree (ln likelihood −5,047.5272) resulting from the analysis of ITS sequences. Isolates in red are subject of this study. Red stars indicate isolates whose draft genomes were *de novo* assembled as part of this work. ITS sequences from type, ex-type, or holotype specimens are included for taxonomic validation. Amplification of portions of the 18S and 28S ribosomal DNA (rDNA) including the intervening internal transcribed spacer regions and 5.8S rDNA (ITS1–5.8S–ITS2) were performed using the primer set ITS1 and ITS4. Maximum likelihood (ML) analysis was performed in MEGA v. 6 by first identifying the best-fit model of nucleotide evolution based on the Akaike Information Criterion. The ML analysis utilized the Nearest-Neighbor-Interchange heuristic search method and topological support was assessed by 1,000 bootstrap replicates. Numbers represent maximum likelihood bootstrap values from 1,000 replicates. Scale bar represents the number of substitutions per site. Additional file 2: Table S1. Assembly metrics for the genomes analyzed in this study. Additional file 3: Table S2. Predicted tRNAs for *D. seriata*, *D. ampelina* and *P. chlamydospora* genomes. Additional file 4: Table S3. Analysis of the repetitive fraction of the *D. ampelina*, *D. seriata*, and *P. chlamydospora* genome. Additional file 5: Table S4. Intron, exon and intergenic space sizes in *D. ampelina*, *D. seriata*, *P. chlamydospora*, and *T. minima*. Additional file 6: Figure S2. Scatter plot showing the relation between the median size of the intergenic space in each scaffold and scaffold length. The lack of correlation between scaffold size and intergenic space suggest that protein coding genes are uniformly distributed in the genomes. Additional file 7: Figure S3. Transcriptome sequencing and gene prediction in *T. minima*. (A) Diagram describing the pipeline used for gene prediction. (B) Comparison of the total number of curated uniprot, *S. cerevisiae,* and *B. cinerea* proteins that matched the first (ab initio only, V1) and the second (V2) versions of the *T. minima* proteomes (BLASTP, *e*-value < 1e^−6^). (C) Density distribution of the alignment coverage of curated uniprot, *S. cerevisiae,* and *B. cinerea* proteins matching proteins in the V1 and V2 *T. minima* predicted proteomes (BLASTP, *e*-value < 1e^−6^ ). (D) Density distribution of the similarity between curated uniprot, *S. cerevisiae,* and *B. cinerea* proteins matching proteins in the V1 and V2 *T. minima* predicted proteomes (BLASTP, *e*-value < 1e^−6^ ). Additional file 8: Table S5. RNA-seq sequencing and mapping statistics. Additional file 9: Table S6.
*Togninia minima* protein sequences. Additional file 10: Table S7. Protein coding gene annotations of all ten fungal species and gene family membership based on TribeMCL clustering. Additional file 11: Figure S4. Scatterplots showing weak correlation between transcriptome size and the number of genes encoding P450s, peroxidases, CAZymes, and proteins involved in secondary metabolism. Additional file 12: Figure S5. Annotated CAZymes in the ten genomes. (A) Barplot showing the total counts of genes in each CAZy class. (B) Projections of the ten fungal species on principal components 1 and 2 based on PCA of CAZy family sizes. Only vectors of the largest loadings are shown. (C) Barplot showing the total counts of AA genes in the ten species. Additional file 13: Table S8. Counts of genes in each genome encoding secreted CAZYmes, P450s, proteins involved in secondary metabolism and peroxidases. Additional file 14: Figure S6. Annotated secondary metabolism gene clusters in the ten genomes. (A) Barplot showing the total counts of genes identified for each cluster type in each fungal species. (B) Projections of the ten fungal species on principal components 1 and 2 based on PCA of abundance of genes associated with each cluster type. Only vectors of the largest loadings are shown. Additional file 15: Figure S7. Boxplots showing the size distribution of the secondary metabolism gene clusters in the ten fungal species. Additional file 16: Table S9. Results of CAFE analysis. Additional file 17: Table S10. Description of the genes in the 90 gene families that are significantly expanded in the Ascomycete trunk pathogens based on CAFE. Additional file 18: Table S11. Functional enrichment based on hypergeometric test in the set of expanded gene families in Ascomycete and Basidiomycete trunk pathogens, and *B. cinerea*. Additional file 19: Table S12. Proteins used to create the clock-calibrated tree in BEAST. Additional file 20: Text S1. BEAST XML input file. Additional file 21: Figure S8. Consensus clock-calibrated phylogenetic tree generated using BEAST. Posterior probabilities are shown. Additional file 22: Table S13. Estimated Sample Sizes (ESS) computed by Tracer.
