# Supplementary figures and images for: Distinctive expansion of gene families associated with plant cell wall degradation, secondary metabolism, and nutrient uptake in the genomes of grapevine trunk pathogens
Source: BMC Genomics. 2015 Jun 19;16(1):469. doi: 10.1186/s12864-015-1624-z (PMC4472170; doi:10.1186/s12864-015-1624-z)

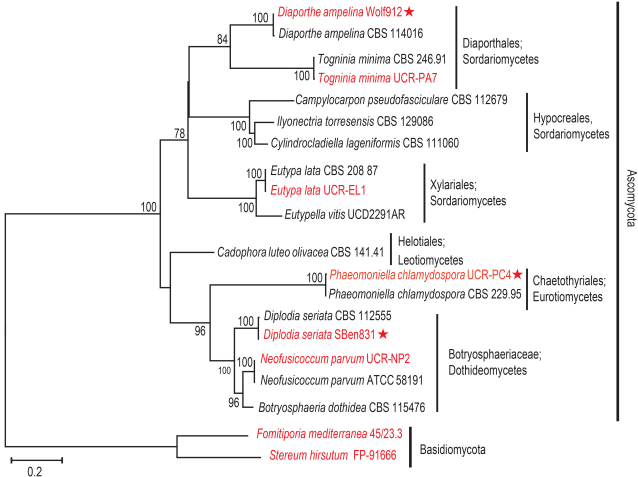

Supplement: Additional file 1: Figure S1. — Single most likely phylogenetic tree (ln likelihood −5,047.5272) resulting from the analysis of ITS sequences. Isolates in red are subject of this study. Red stars indicate isolates whose draft genomes were de novo assembled as part of this work. ITS sequences from type, ex-type, or holotype specimens are included for taxonomic validation. Amplification of portions of the 18S and 28S ribosomal DNA (rDNA) including the intervening internal transcribed spacer regions and 5.8S rDNA (ITS1–5.8S–ITS2) were performed using the primer set ITS1 and ITS4. Maximum likelihood (ML) analysis was performed in MEGA v. 6 by first identifying the best-fit model of nucleotide evolution based on the Akaike Information Criterion. The ML analysis utilized the Nearest-Neighbor-Interchange heuristic search method and topological support was assessed by 1,000 bootstrap replicates. Numbers represent maximum likelihood bootstrap values from 1,000 replicates. Scale bar represents the number of substitutions per site. [file 12864_2015_1624_MOESM1_ESM.pdf]

*T. minima*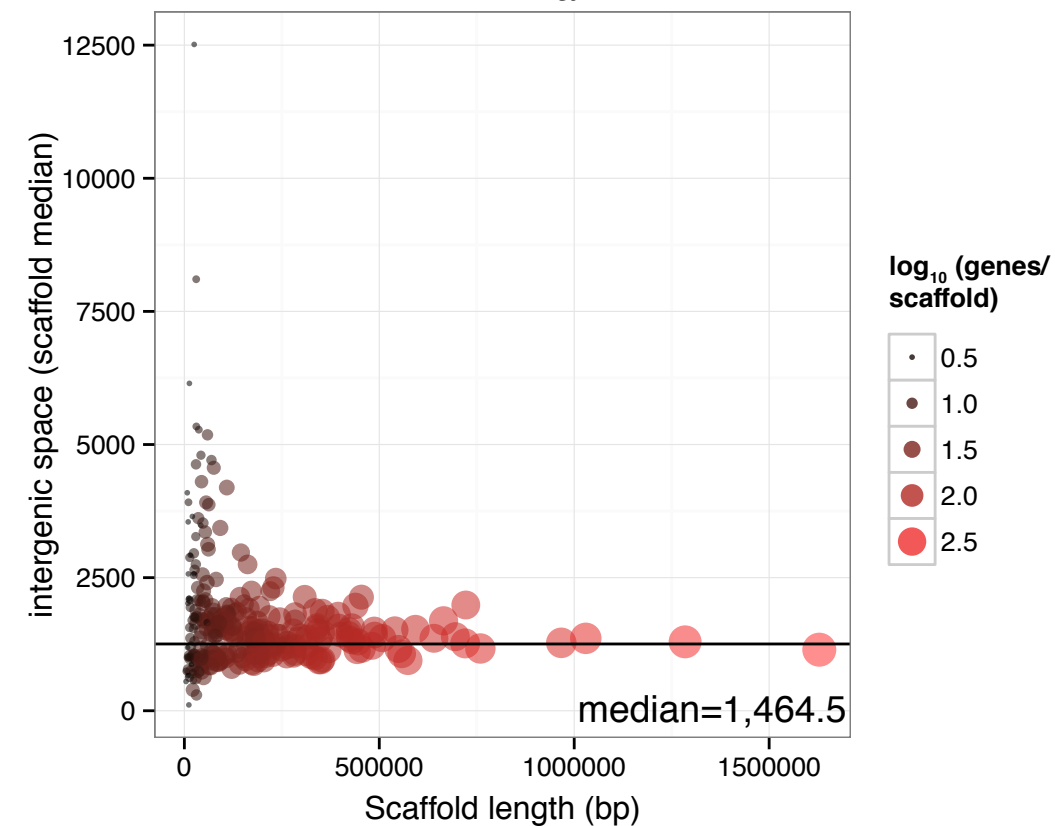*D. seriata*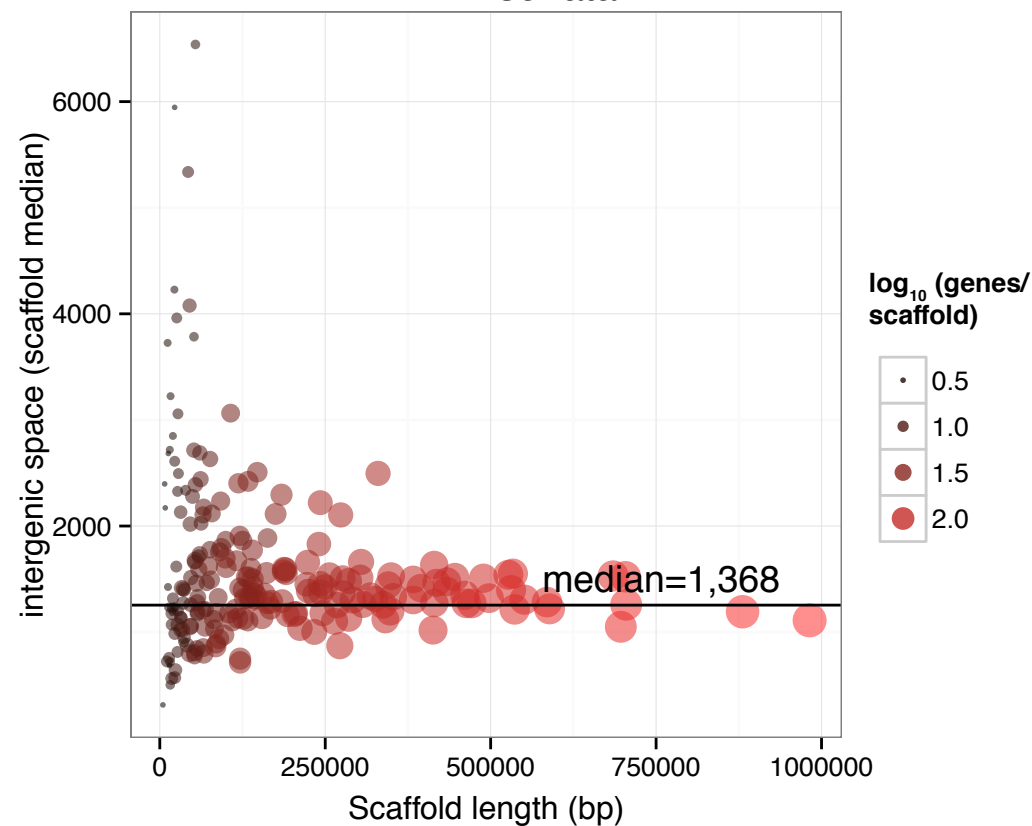*D. ampelina*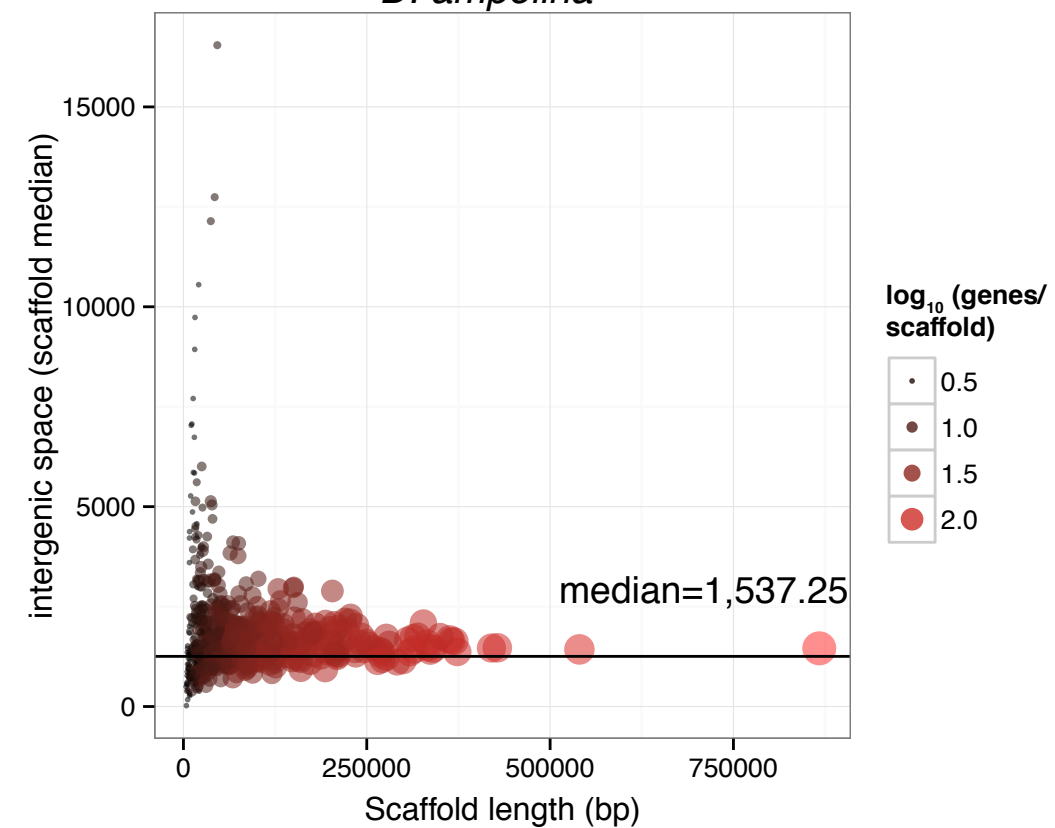*P. chlamydospora*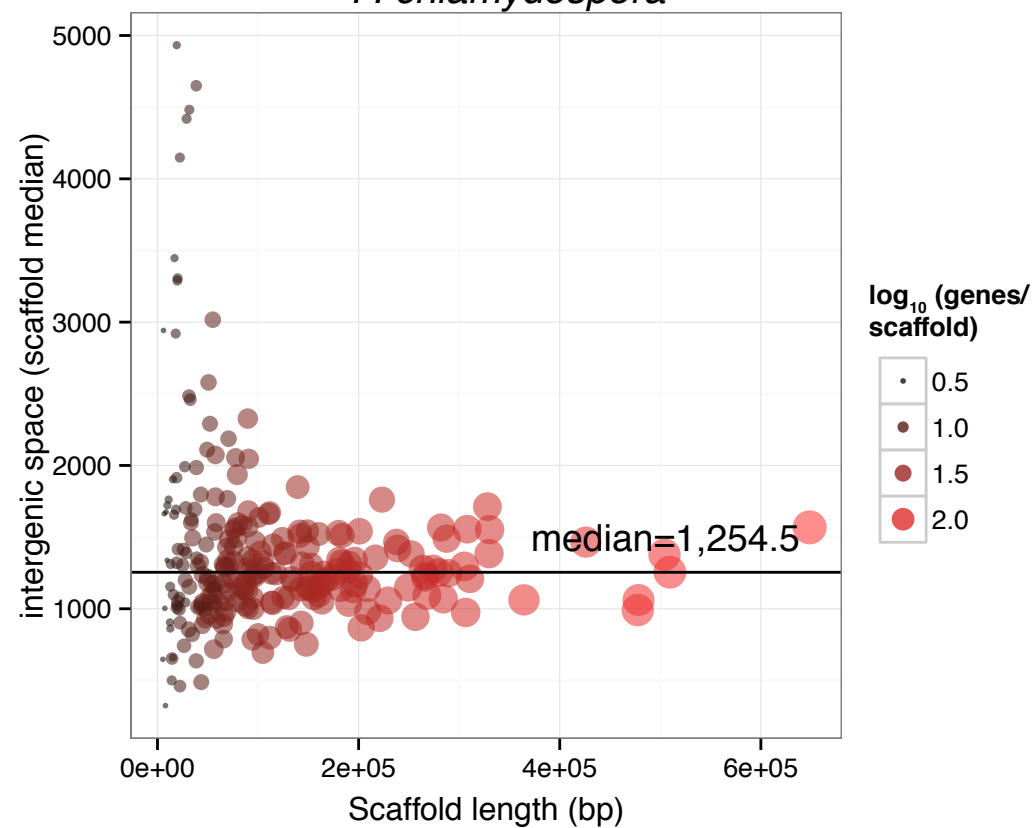

Supplement: Additional file 6: Figure S2. — Scatter plot showing the relation between the median size of the intergenic space in each scaffold and scaffold length. The lack of correlation between scaffold size and intergenic space suggest that protein coding genes are uniformly distributed in the genomes. [file 12864_2015_1624_MOESM6_ESM.pdf]

**A**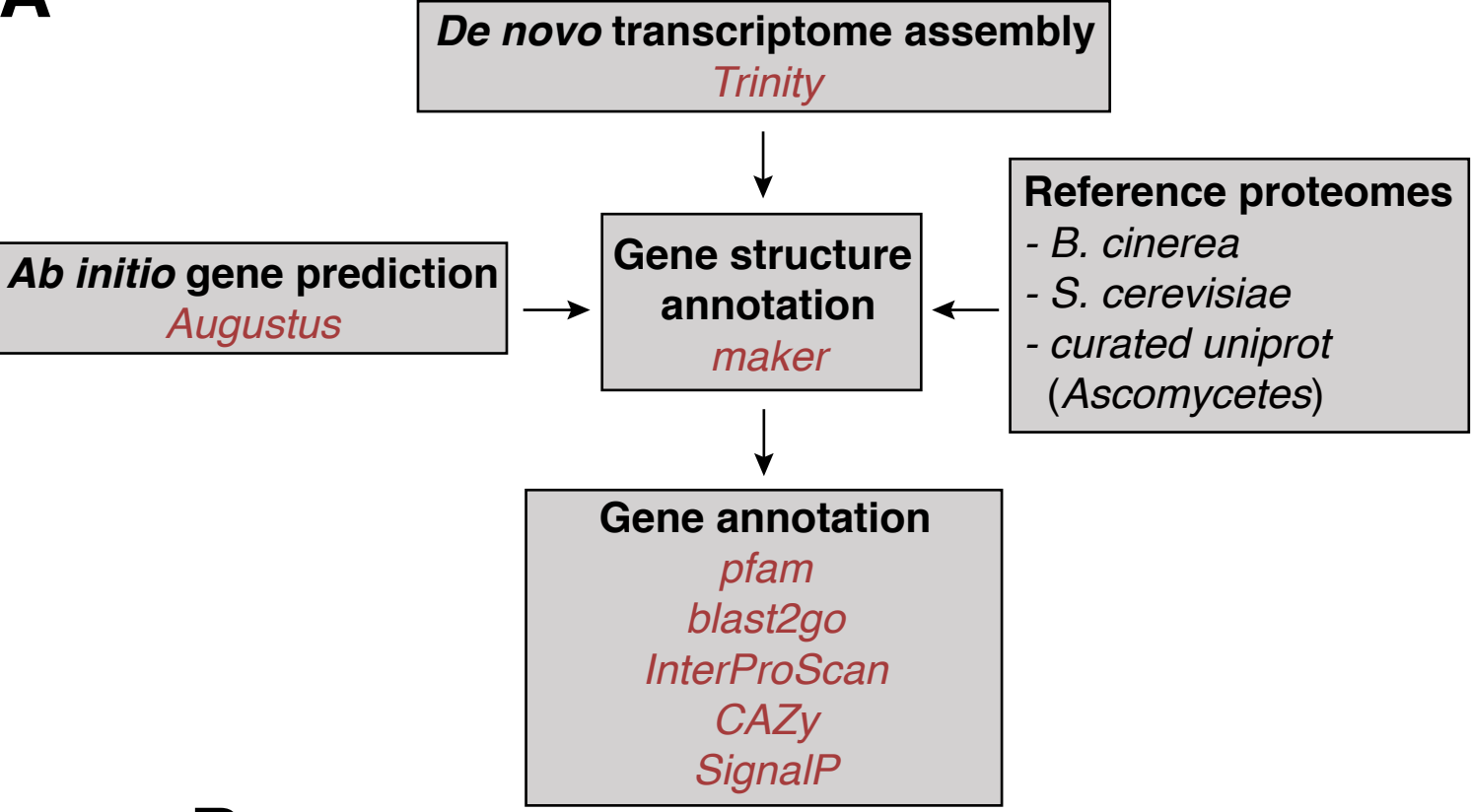**B**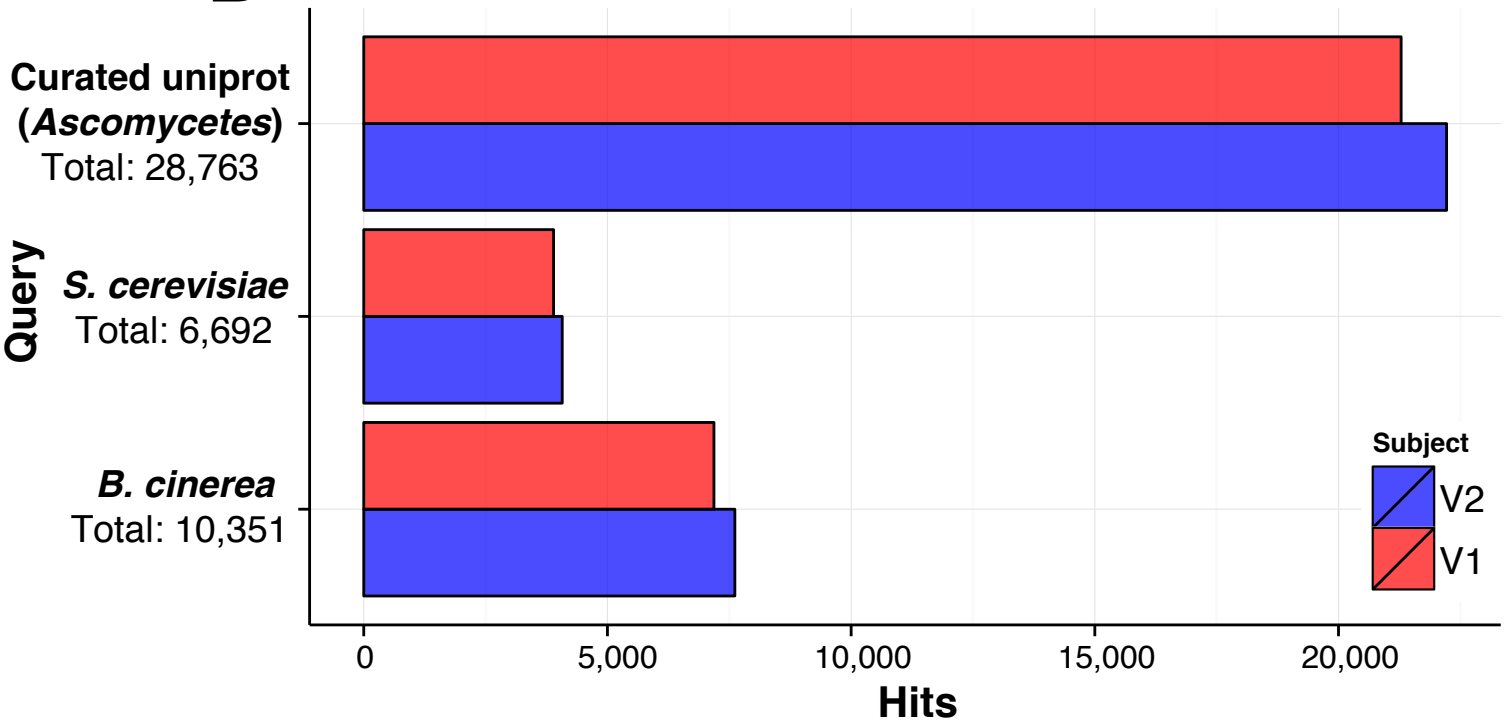**C**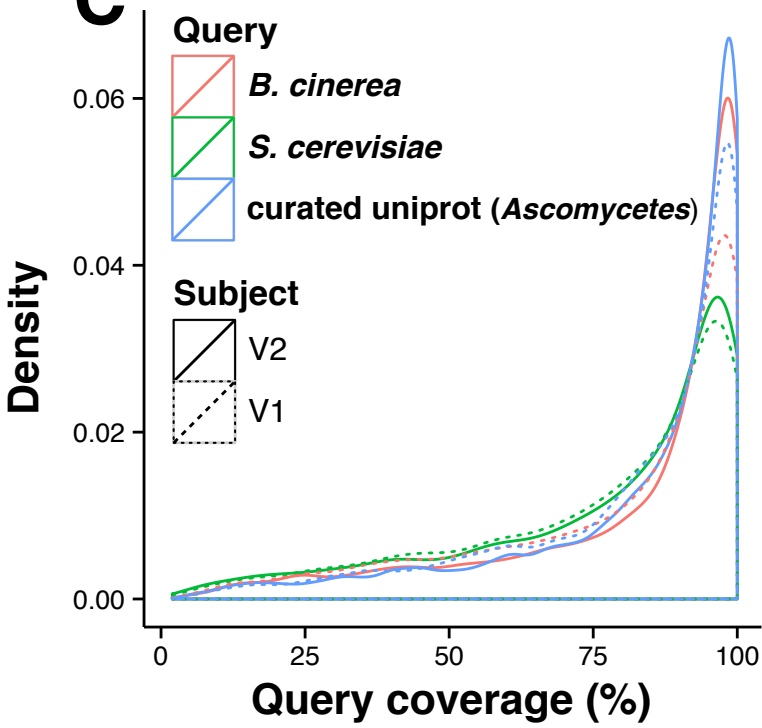**D**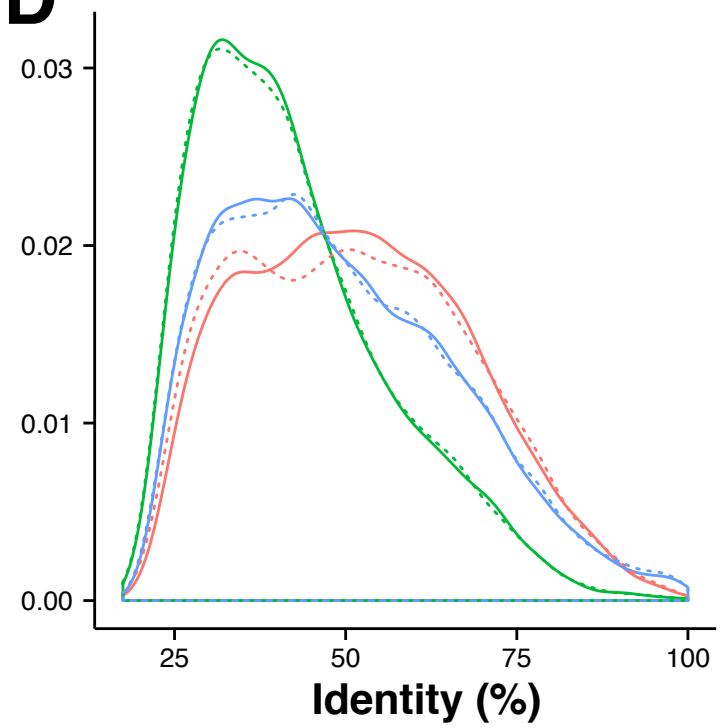

Supplement: Additional file 7: Figure S3. — Transcriptome sequencing and gene prediction in T. minima. (A) Diagram describing the pipeline used for gene prediction. (B) Comparison of the total number of curated uniprot, S. cerevisiae, and B. cinerea proteins that matched the first (ab initio only, V1) and the second (V2) versions of the T. minima proteomes (BLASTP, e-value < 1e−6). (C) Density distribution of the alignment coverage of curated uniprot, S. cerevisiae, and B. cinerea proteins matching proteins in the V1 and V2 T. minima predicted proteomes (BLASTP, e-value < 1e−6 ). (D) Density distribution of the similarity between curated uniprot, S. cerevisiae, and B. cinerea proteins matching proteins in the V1 and V2 T. minima predicted proteomes (BLASTP, e-value < 1e−6 ). [file 12864_2015_1624_MOESM7_ESM.pdf]

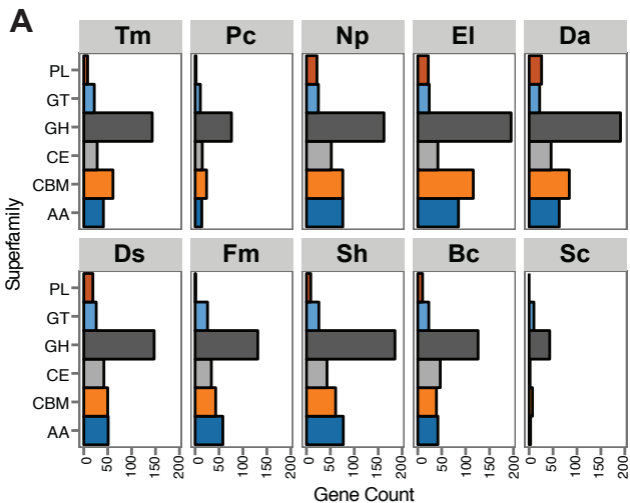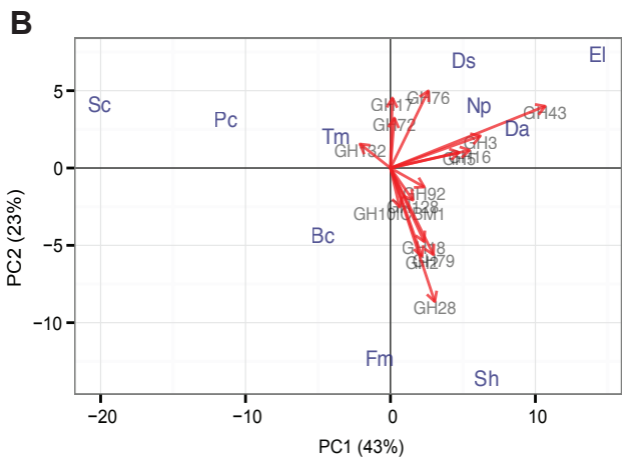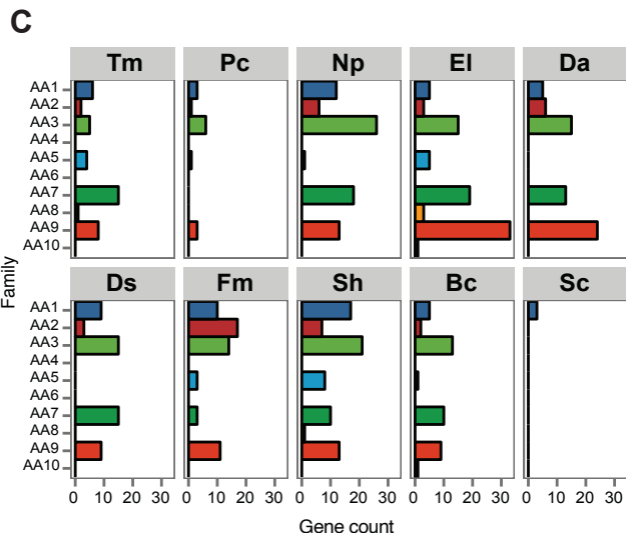

Supplement: Additional file 12: Figure S5. — Annotated CAZymes in the ten genomes. (A) Barplot showing the total counts of genes in each CAZy class. (B) Projections of the ten fungal species on principal components 1 and 2 based on PCA of CAZy family sizes. Only vectors of the largest loadings are shown. (C) Barplot showing the total counts of AA genes in the ten species. [file 12864_2015_1624_MOESM12_ESM.pdf]

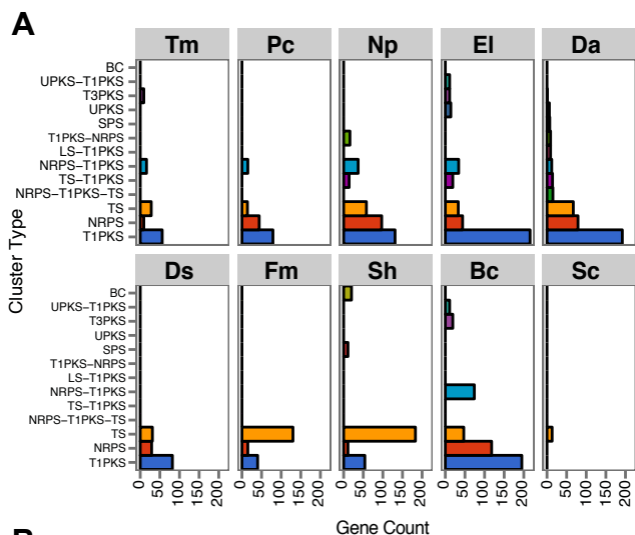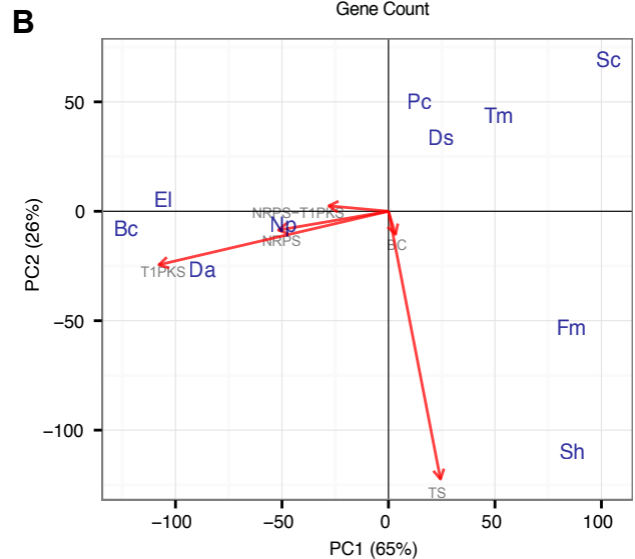

Supplement: Additional file 14: Figure S6. — Annotated secondary metabolism gene clusters in the ten genomes. (A) Barplot showing the total counts of genes identified for each cluster type in each fungal species. (B) Projections of the ten fungal species on principal components 1 and 2 based on PCA of abundance of genes associated with each cluster type. Only vectors of the largest loadings are shown. [file 12864_2015_1624_MOESM14_ESM.pdf]

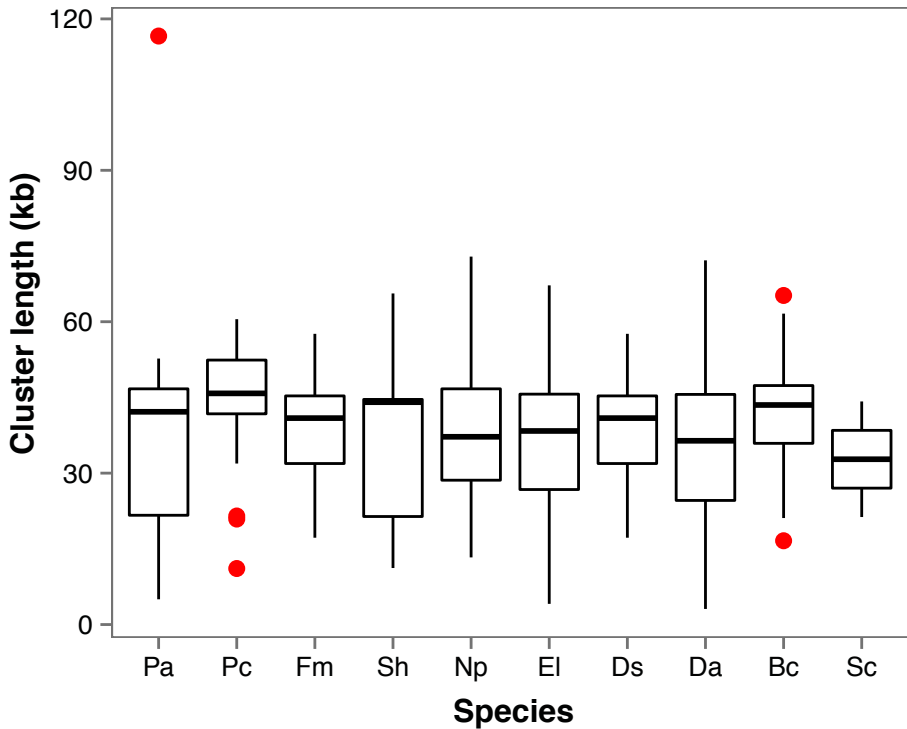

Supplement: Additional file 15: Figure S7. — Boxplots showing the size distribution of the secondary metabolism gene clusters in the ten fungal species. [file 12864_2015_1624_MOESM15_ESM.pdf]

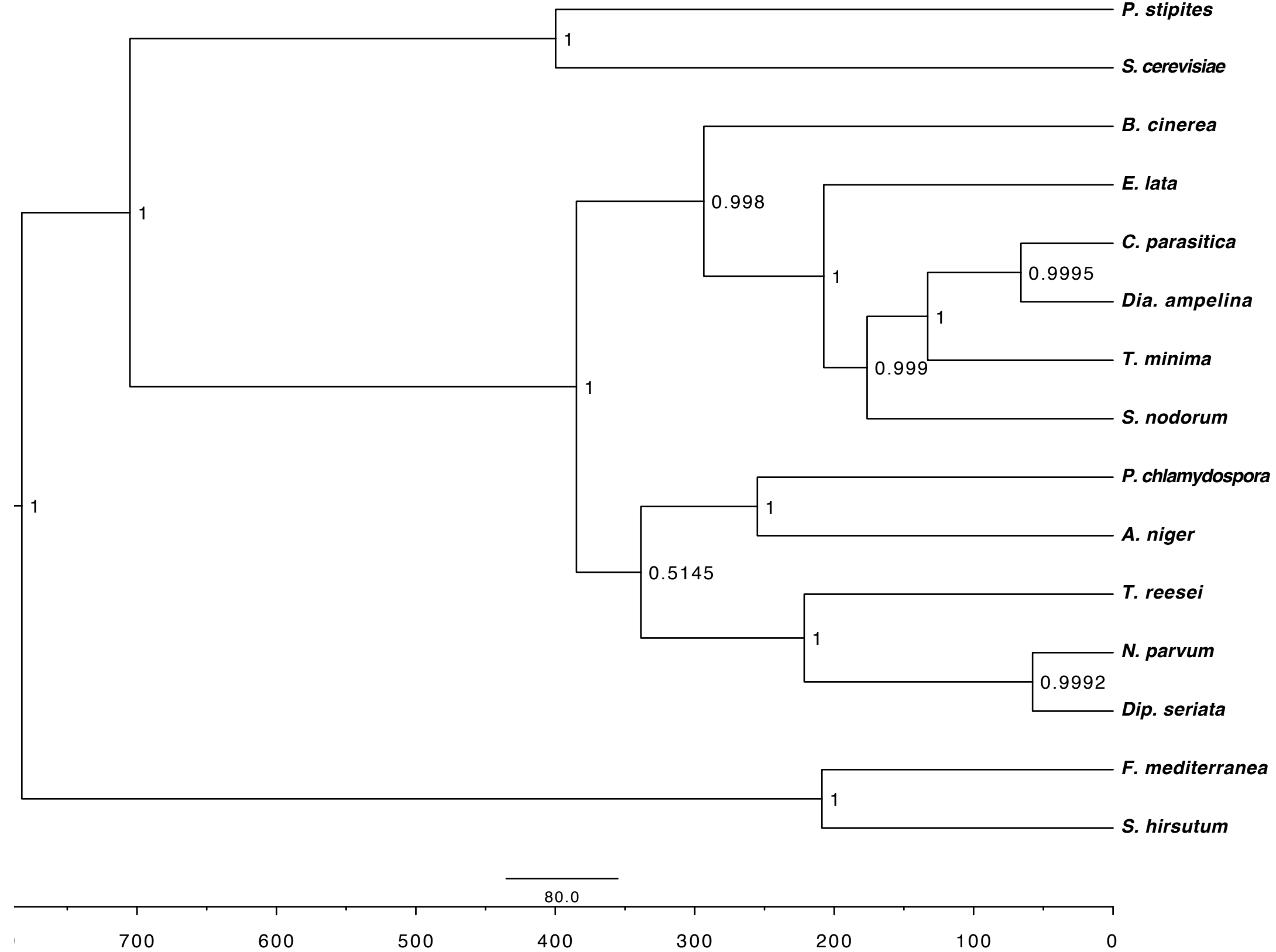

Supplement: Additional file 21: Figure S8. — Consensus clock-calibrated phylogenetic tree generated using BEAST. Posterior probabilities are shown. [file 12864_2015_1624_MOESM21_ESM.pdf]
