# Supplementary material for: Distinctive expansion of gene families associated with plant cell wall degradation, secondary metabolism, and nutrient uptake in the genomes of grapevine trunk pathogens
Source: BMC Genomics. 2015 Jun 19;16(1):469. doi: 10.1186/s12864-015-1624-z (PMC4472170; doi:10.1186/s12864-015-1624-z)
Supplement: Additional file 11: Figure S4. — Scatterplots showing weak correlation between transcriptome size and the number of genes encoding P450s, peroxidases, CAZymes, and proteins involved in secondary metabolism. [file 12864_2015_1624_MOESM11_ESM.pdf]

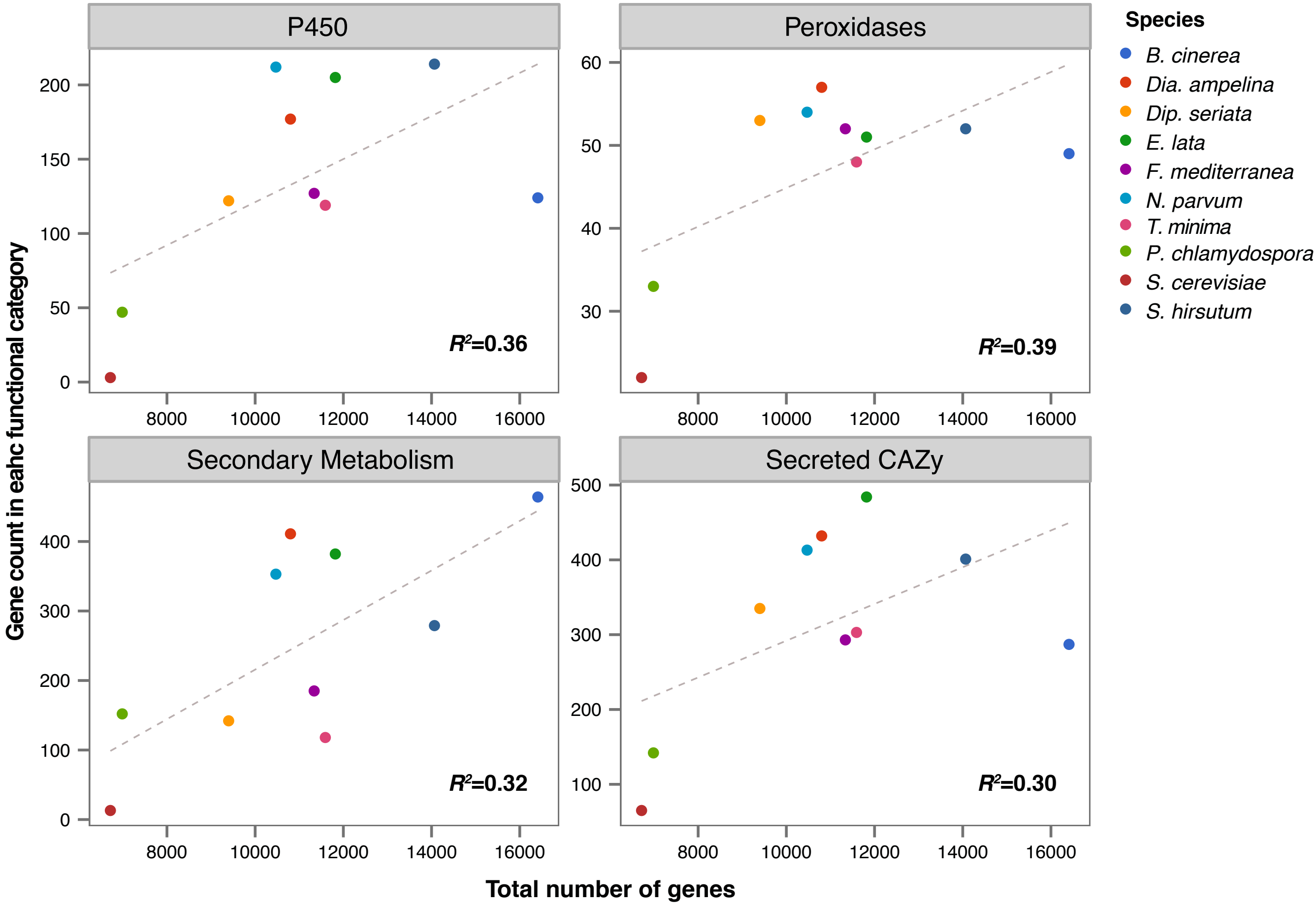
P450
Peroxidases
Secondary Metabolism
Secreted CAZy
R²=0.36
R²=0.39
R²=0.32
R²=0.30
Gene count in eahc functional category
Total number of genes
Species
B. cinerea
Dia. ampelina
Dip. seriata
E. lata
F. mediterranea
N. parvum
T. minima
P. chlamydospora
S. cerevisiae
S. hirsutum
